# REDAC: RNA-seq expression data analysis chatbot

**DOI:** 10.1093/bioadv/vbaf321

**Published:** 2025-12-27

**Authors:** Giovanni Maria De Filippis, Pranoy Sahu, Pasqualina Ambrosio, Stefania Picascia, Matteo Lo Monte, Ilenia Agliarulo, Simone Di Paola, Cristiano Russo, Christian Tommasino, Nicola Normanno, Daniela Frezzetti, Seetharaman Parashuraman, Antonio M Rinaldi, Francesco Russo

**Affiliations:** Department of Electrical Engineering and Information Technology, University of Naples Federico II, Naples, 80125, Italy; Consiglio Nazionale Delle Ricerche, Naples, 80131, Italy; Consiglio Nazionale Delle Ricerche, Naples, 80131, Italy; Consiglio Nazionale Delle Ricerche, Naples, 80131, Italy; Consiglio Nazionale Delle Ricerche, Naples, 80131, Italy; Consiglio Nazionale Delle Ricerche, Naples, 80131, Italy; Consiglio Nazionale Delle Ricerche, Naples, 80131, Italy; Department of Electrical Engineering and Information Technology, University of Naples Federico II, Naples, 80125, Italy; Department of Electrical Engineering and Information Technology, University of Naples Federico II, Naples, 80125, Italy; IRCCS Istituto Romagnolo per lo studio dei Tumori “Dino Amadori” (IRST), Meldola, 47014, Italy; Cell Biology and Biotherapy Unit, Istituto Nazionale Tumori-IRCCS-Fondazione G. Pascale, Naples, 80131, Italy; Consiglio Nazionale Delle Ricerche, Naples, 80131, Italy; Department of Electrical Engineering and Information Technology, University of Naples Federico II, Naples, 80125, Italy; Consiglio Nazionale Delle Ricerche, Naples, 80131, Italy

## Abstract

**Motivation:**

To date, due to the complexity of both the analytical processes and the result interpretation of RNA-seq expression data analyses, researchers often require the support of bioinformaticians expertise. Selecting appropriate statistical tests and performing essential data manipulations, such as normalization and filtering, in a rigorous and reproducible manner remains a significant challenge for many users.

**Results:**

We developed REDAC, a web-based R application that offers an interactive platform designed to simplify and enhance RNA-seq expression data exploration and analysis. REDAC provides a straightforward approach to perform differentially RNA-seq analysis rapidly, easily, and transparently through natural language queries from users. Moreover, it allows to run complete analyses, generate comprehensive visualizations, and obtain biological interpretation of pathway enrichment results via two popular Large Language Models: *Gemma* and *LLaMA* guided by a PubMed based Retrieval-Augmented Generation module. Finally, REDAC promotes reproducibility through the automated generation of analysis reports.

**Availability and implementation:**

REDAC is available for local (https://github.com/franruss/REDAC) and online use (https://frusso.shinyapps.io/REDAC). User manual: https://github.com/franruss/REDAC/blob/main/docs/REDAC_user_manual.pdf

## 1 Introduction

Differential expression analysis (DEA) (Rosati *et al.* 2024) is a crucial process in the analysis of RNA-seq data to identify gene expression variations under different experimental conditions or treatments in biological systems, including cell lines, tissues, organoids, spheroids, and patient biopsies. This analysis serves as a critical first step in establishing correlations between transcriptional changes and phenotypic alterations in the biological model under investigation.

Gene expression analysis plays an essential role in multiple areas of biomedical research, including biomarker discovery (e.g. gene signature) (Sheng *et al.* 2020), characterization of drug-response-associated genes (Liu *et al.* 2016), drug repurposing studies (Dave *et al.* 2024), assessment of drug-induced toxicity (Cui and Paules 2010), genetic association studies (Pividori *et al.* 2023), elucidation of disease mechanisms (Kolobkov *et al.* 2022) and in the development of new therapeutic approaches (Garcia-Milian *et al.* 2018).

The standard RNA-seq analysis workflow, beginning with raw count expression data (discrete, non-negative integer values), comprises several steps according to established protocols (Corchete *et al.* 2020). The initial preprocessing phase involves normalization (Li *et al.* 2015) and filtering processing of low-expression genes (Ying Sha *et al.* 2015), since RNA-seq data are subject to various technical biases, including sequencing depth variations, gene length effects, and other systematic distortions (Finotello *et al.* 2012). Multiple normalization methods are available for each type of distortion, including: *TMM* (Singh *et al.* 2024), *UQUA* (Bullard et al. 2010), *FQUA* (Bolstad *et al.* 2003), and *RPKM* (Mortazavi *et al.* 2008).

The second step accounts for the application of an appropriate statistical test for differential analysis of gene expression under experimental conditions. The most popular methods available are: *DESeq2* (Love *et al.* 2014), *edgeR* (Robinson *et al.* 2010), and *limma* (Ritchie *et al.* 2015). Finally, results interpretation is facilitated by the examination of diagnostic plots that reveal different aspects of the expression patterns, such as *volcano* plots, *MA* plots, *dot* plots, *heatmaps*.

To support non-expert users in this analytical workflow, several platforms and graphical user interfaces (GUIs) have been developed to automate RNA-seq analysis, including: *RNASeqGUI* (Russo and Angelini 2014, Russo *et al.* 2016a), *Galaxy* (Batut *et al.* 2021), and *GEO2R* (Clough *et al.* 2024). However, these tools present significant limitations. Users require a comprehensive understanding of the underlying computational steps to minimize procedural errors, while the analytical accuracy of these applications remains critical to obtain reliable results and avoiding misleading biological interpretations. Consequently, performing DEA in an expert-independent manner remains challenging and requires substantial programming expertise, statistical knowledge and data manipulation skills (Mikalef *et al.* 2018).

## 2 RNA-seq analysis chatbots and LLM-based tools

The shortage of expert RNA-seq data analysts represents a significant barrier to research advancement in this field. Support for DEA result interpretation can provide crucial insight for understanding the underlying biological mechanisms. Therefore, developing a comprehensive pipeline for accurate data representation (plot generation), manipulation (filtering, normalizations, transformations), and interpretation (information and features extraction) is fundamental for determining whether data support the pre-stated hypotheses. To this end, appropriate use of Large Language Models (LLMs) could significantly assist both analysis and interpretation processes.

Unfortunately, several challenges prevent the development of an accurate and complete data analysis pipeline. Most LLMs have shown promising results in short code generation only for simple, isolated tasks (Piccolo et al. 2023). However, for complex or more structured data analysis tasks, the LLM-generated code usually contains several errors (both conceptual and syntactic) and requires expert supervision to ensure syntactically correct and conceptually accurate code for execution, with appropriate statistical test selection based on the data characteristics. Recently, some Bio-chatbot systems have been developed to address these challenges while enabling users to perform focused analytical tasks. Some efforts have been made to exploit LLMs in the areas of expert analysts. Bioinformatics applications, such as *DrBioRight* (Liu *et al.* 2025), *BioMANIA* (Dong *et al.* 2024), *BIA* (Bio-Informatics Agent) (Xin *et al.* 2024), and *AutoBA* (Auto Bioinformatics Analysis) (Zhou *et al.* 2024) represent well-designed examples of this attempt to exploit LLMs for the execution of multiple bioinformatics tasks.

**DrBioright** is suitable for biomedical researchers with limited programming experience and provides an intuitive workflow for common bioinformatics tasks, displaying “Chain of Thoughts” explanations during the execution. However, it is and does not offer alternative analytical approaches through interactive discussions or alternative code chunks in R. Moreover, advanced statistical modelling (e.g. filtering processes that minimize statistical power loss or data normalizations that reduce unwanted distortions in the RNA-seq data) is not supported. Furthermore, reproducibility is compromised as the underlying code remains not accessible. Finally, this chatbot exhibits a strong dependence on external database resources, cannot efficiently handle large datasets, and does not provide alternative models, tests, or code chunks that would benefit expert analysts.

**BioMANIA** is an AI-driven, natural language-oriented platform designed to simplify bioinformatics data analysis. By integrating LLMs with bioinformatics tools, BioMANIA enables users to execute analyses through simple queries. The main advantages lie in the automatic generation of well-documented Python-based tools through the creation of synthetic instructions. Moreover, it provides an assistant that chooses the appropriate analysis. Currently, it exclusively supports Python-based tools only and requires high GPU computational resources to run locally.

**BIA** automatically generates workflows by extracting pipelines from the scientific literature, facilitating tool integration, and reducing manual setup requirements. It supports the processing of raw data and metadata from public databases and generates code chunks for various analyses, enabling non-expert users to perform sophisticated analyses. Moreover, BIA exclusively focuses on Python-based tools.

**AutoBA** generates detailed step-by-step analytical plans, executes code and performs data analysis. It accepts multiple omics datasets as input, including whole genome sequencing (WGS), single-cell RNA-seq, ChIP-seq, and spatial transcriptomics. It supports both local and online deployment while ensuring data security. Moreover, extensive GPU computational resources are needed to run locally.

Despite these advances, existing LLM-based bioinformatics systems exhibit several critical limitations that constrain their practical utility. Most importantly, these platforms rely predominantly on predefined, standardized analytical workflows, which restrict their applicability across diverse experimental scenarios. When tasked with generating R code for complex analytical requirements—including multi-factorial experimental designs, custom normalization procedures, advanced filtering strategies, or specialized visualization approaches—these systems frequently produce incomplete or syntactically incorrect code that requires an expert-level correction and validation. Additionally, the lack of consensus on standardized data analysis pipelines further complicates the development of robust, generalizable solutions.

## 3 REDAC architecture

To address all these limitations, we introduce REDAC (RNA-seq Expression Data Analysis Chatbot), a comprehensive web-based platform designed to support both novice and expert users engaged in RNA-seq data analysis. The platform integrates two state-of-the-art LLMs: Gemma (Gemma Team 2024), developed by Google (version: gemma-3n-E4B-it) and LLaMA (Touvron *et al.* 2023), developed by Meta (version: Llama-3.3-70B-Instruct-Turbo).

The core features of REDAC include:

- **Standardized data analysis pipelines:** REDAC can execute standard, pre-implemented *edgeR*-based (Robinson *et al.* 2010) workflows suitable for users requiring rapid or reproducible analyses without programming expertise. Simultaneously, it generates modular, customizable R code snippets tailored for advanced analytical scenarios. This dual-mode architecture enables experienced bioinformaticians to explore alternative statistical models, implement custom contrast testing, and seamlessly integrate *edgeR* output into broader R-based analytical frameworks.
- **Management of hallucinations:** REDAC mitigates the occurrence of hallucinations, which are a recognized limitation of most current platforms. This improvement is primarily due to its interaction model, in which users submit queries in natural language using a conversational interface. These queries are processed by Gemma and LLaMA, which are explicitly constrained to generate output exclusively in JSON format. The structured output adheres to a predefined schema specifying the required data structure and parameters, thereby enforcing syntactic consistency, enabling robust automated downstream processing, and significantly reducing the risk of misinterpretation.
- **Interpretation of enrichment results via a RAG module**: A distinguishing feature of REDAC is its dual support for interpreting enrichment analysis results. Upon request for interpretative assistance, both Gemma and LLaMA are asked to assume the role of expert bioinformaticians, providing context aware explanations of the biological mechanisms underlying the observed enrichment patterns. In contrast to traditional tools that usually present a static list of pathways, REDAC leverages these LLMs to synthesize insights across the entire set of enriched pathways and suggest biologically meaningful directions for further investigation. In particular, the interpretation of enrichment results by LLMs is implemented through a RAG module. Users are prompted to provide a concise yet detailed description of the experimental design and the corresponding input data. This textual input is processed to extract informative keywords, which are subsequently employed to query PubMed website (https://pubmed.ncbi.nlm.nih.gov) and retrieve a collection of contextually relevant scientific articles. The keyword extraction procedure involves filtering out non-specific or common terms while retaining relevant entities, including drug names, cell lines, species, treatments, and disease identifiers. The resulting literature corpus constitutes a biologically grounded knowledge base that supports the generation of context aware interpretations by the LLMs. The richer and more specific the user prompt, the more accurate and meaningful the resulting interpretations. Therefore, the interpretation is biologically grounded because it is guided and addressed by the following four sources of information. (i) user’s description (in which the user can describe the context and the experiment performed), (ii) most significant genes, (iii) list of enriched pathways, and (iv) scientific papers downloaded from PubMed (based on keywords extracted from the user description). These four sources of information contribute together to generate the RAG module. All referenced articles used to build the RAG module are explicitly cited. We hope this approach enhances the ability to generate meaningful and well-structured summaries organized by biological categories, including mechanistic hypotheses and suggestions for future investigations.
- **Intuitive conversational interface:** REDAC enables researchers to conduct complex analyses through natural language queries, eliminating the need for programming expertise. The platform is implemented in R using *shiny* (Chang *et al.* 2025) and *plotly* (Sievert 2020) frameworks to deliver a responsive, interactive user experience. The interface comprises three functionally distinct modules, each accessible through dedicated tabs to streamline user workflows.
- **Reproducibility and transparency:** Upon completion of the analysis, REDAC automatically generates comprehensive documentation in dual formats: static Word documents suitable for Supplementary Materials, available as supplementary data at *Bioinformatics Advances* online, and interactive HTML reports for dynamic exploration. This automated reporting functionality ensures complete methodological transparency, enables immediate quality assessment, and promotes reproducible research practices (Russo *et al.* 2016b) of the entire analytical pipeline.

The architecture of the REDAC system is structured as a modular framework comprising three core functional components, each responsible for distinct aspects of RNA-seq data processing. The architecture promotes the separation of concerns through loosely coupled modules that facilitate efficient workflow management. Each component provides well-defined interfaces and is described in the following in terms of functionality and technical implementation.

**Figure 1.**
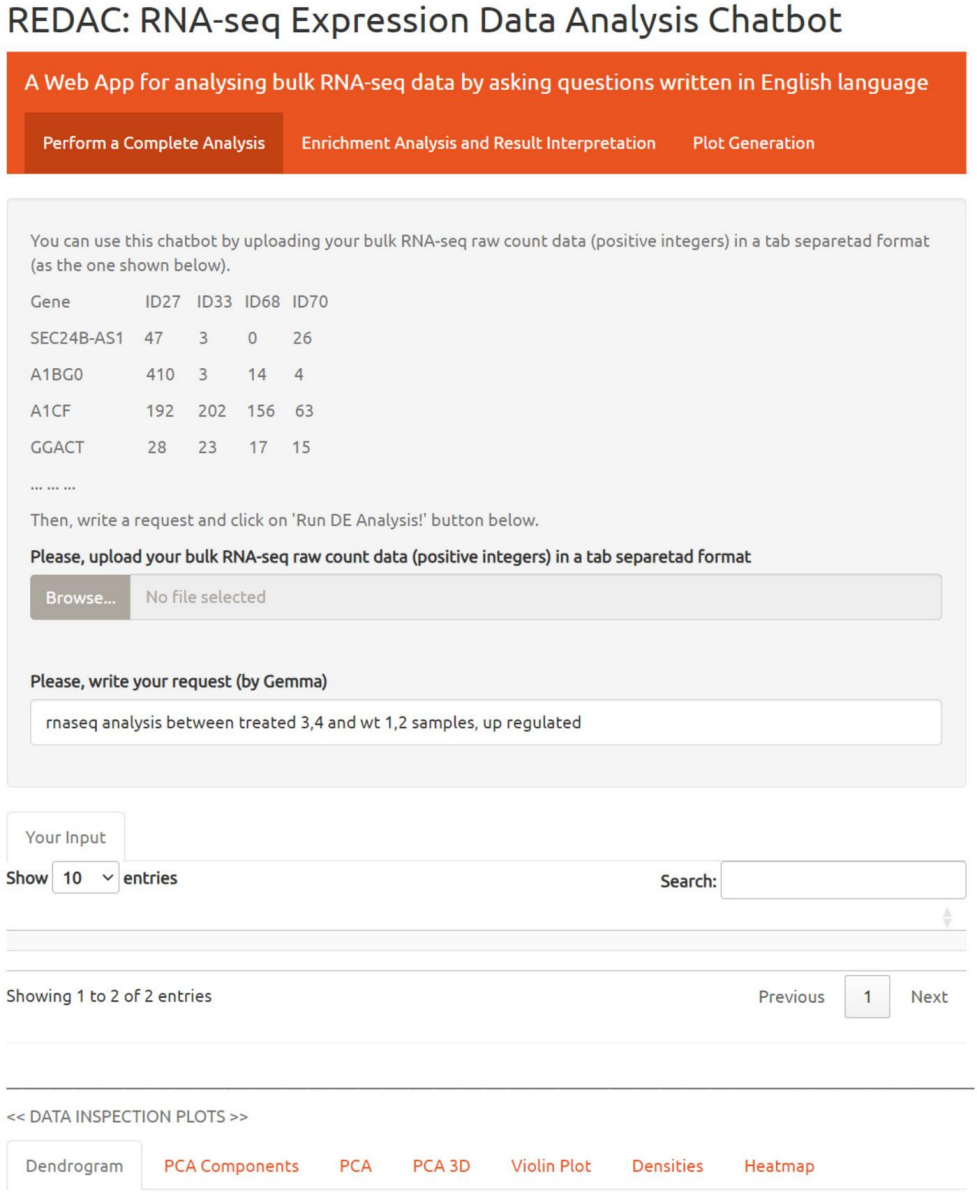
REDAC main interface.

The user interface shown in Fig. 1 constitutes the Perform a Complete Analysis module, guiding users through a complete RNA-seq differential expression workflow using raw count data as input via natural language interaction with Gemma LLM. This module implements a standardized *edgeR*-based analytical pipeline and generates an extensive suite of diagnostic and interpretive visualizations, including: variance component histograms quantifying principal component contributions, two- and three-dimensional PCA projections, violin plots for distribution assessment, density plots for distributional characterization, hierarchical clustering dendrograms illustrating sample similarities (Ward’s linkage with Euclidean distance), and expression heatmaps displaying patterns across the 1000 most variable genes. These visualizations facilitate data quality assessment, outlier detection, and batch effect identification. Following quality control inspection and correction of potential systematic errors (e.g. sample labeling discrepancies), users initiate the analysis via the *Run Analysis!* interface. Upon completion, volcano plots and MA plots (where M represents log2 fold change and A denotes log2 average expression intensity) enable a comprehensive inspection of differential expression results. In addition to the list of significantly regulated genes (differentially expressed, up-regulated, or down-regulated), this module provides two specialized features: Analysis Discussion (by *Gemma*), designed for novice users and providing structured explanations of standard RNA-seq analytical workflows with detailed step-by-step methodology descriptions, and Alternative code for R developers (by *LLaMA*), targeting experienced programmers with customizable R code segments for advanced statistical modeling and alternative analytical approaches directly within the R environment.

The Enrichment Analysis and Result Interpretation module enables comprehensive pathway enrichment analysis through integration with 16 different databases for functional annotation of differentially expressed gene sets. Moreover, in this section, users are encouraged to provide a brief description outlining the experimental context and biological question. This information is used to query PubMed and retrieve relevant literature for constructing the RAG module. This is used by both LLMs to generate context aware interpretations.

The Plot Generation module facilitates rapid exploratory data analysis through natural language interaction with Gemma. It is designed for users who require single interactive visualizations of an input data without performing a complete analysis pipeline. This module provides additional data insights and enables users to generate both standard visualizations (automatically produced in the first module) and specialized plots not available in the previous modules, including *boxplots*, *correlation heatmaps*, *surface plots*, and *network visualizations*.

A comparison of key features between REDAC and existing LLM-based bioinformatics tools is presented in Table 1. REDAC adds several key improvements over existing platforms in the bioinformatics analysis landscape. Unlike tools such as BioMANIA and BIA, which are prone to hallucinations and primarily focus on Python-based workflows, REDAC significantly mitigates these issues through its structured JSON output format and dual-LLM architecture. REDAC uniquely combines the accessibility required by novice users with the advanced customization capabilities demanded by expert bioinformaticians. The platform’s dual-model approach (Gemma and LLaMA) for code generation and interpretation provides multiple analytical perspectives, a feature absent in competing tools. Furthermore, REDAC’s comprehensive visualization suite, including rich interactive plots, surpasses the basic static visualizations offered by most existing platforms. The R-based implementation addresses a significant gap in the current ecosystem, which is dominated by Python-focused chatbots, while maintaining full code transparency and reproducibility through automated report generation—features that are either limited or absent in comparable platforms.

**Table 1.** Comparative table of the most used bioinformatics LLM-based tools.

Feature	DrBioRight	BioMANIA	BIA	AutoBA	REDAC
Target users	Biomedical researchers with coding knowledge	Researchers using Python-based tools	General researchers	Users across multi-omics (low expertise in coding)	Both: novice users and expert bioinformaticians
Interface	Web GUI	Natural language + chatbot UI	Natural language + automated workflows	Simple user interface	Web GUI (shiny + plotly)
Customization	Predefined workflows	Limited to tool documentation	Automated code/workflow generation	Fully automated with minimal input	Predefined + customizable R code generation
Programming language focus	Hidden	Python	Python	Python	R
AI/LLM integration	None	Yes (LLMs for chatbot creation)	Yes (LLM-guided pipeline + reporting)	Yes (LLM for planning + analysis)	Yes (GEMMA and LLaMA for alternative code and interpretations via a PubMed-based RAG module)
Visualization	Basic static plots	Limited; depends on integrated tools	Standard visualizations via auto-code	Auto-generated plots	Rich interactive visualizations (PCA 2D/3D, MA, Volcano, Heatmap, etc.)
Tool adaptability	Fixed pipelines	Depends on tool documentation	Can adapt to literature-based workflows	Adapts to new bioinformatics tools	Fixed pipelines + expert users can modify suggested code
Error handling	Manual correction	Prone to hallucinations	Prone to hallucinations	Includes Auto Code Repair (ACR)	Manual correction by showing the code used in the report
Deployment	Web-based only	Docker-based	Not clearly specified	Local & online	Both: web-based and local
Documentation transparency	Low	Based on API docs	Auto-documents workflows	Generates documentation	Full code transparency for reproducibility

The key benefits for REDAC users are summarized in Table 2.

**Table 2.** Summary of benefits by using REDAC.

Feature	User type	Value
One-click analysis	Non-programmers	No coding skills required
Customizable R code	Bioinformaticians	Full control over pipeline
Interactive plots	All users	Immediate data visualization
Dual LLM interpretations	Biologists	Biological interpretations via a RAG module
Transparent code	All users	Reproducibility

## 4 Case study

To demonstrate the utility and feasibility of REDAC, we analyzed publicly available RNA-seq data from a previously published study (Frezzetti *et al.* 2024). This study investigated the biological mechanisms underlying resistance to suboptimal doses of EGFR-tyrosine kinase inhibitors in Non-Small cell lung cancer (NSCLC) cells. The authors generated Gefitinib-resistant or GR lines (HCC827 and PC9) using different treatment regimens: (i) continuous exposure with fixed or incrementally increasing concentrations and (ii) intermittent exposure. Non-resistant parental cell lines served as controls for comparison with their respective GR derivatives. The raw RNA-seq count data were obtained in tab-separated format to evaluate REDAC’s analytical capabilities.

**Goal**: To demonstrate the analytical capabilities of REDAC using publicly available RNA-seq count data illustrate how the platform benefits both non-programming researchers and expert bioinformaticians.

**Source**: “Raw data RNA-seq for submission with gene name 3 1 24.xlsx” available at https://zenodo.org/records/11057181

**Description**: The data set comprises RNA-seq count data generated via paired-end sequencing on the Illumina NextSeq 500 platform from 16 samples representing two non-small cell lung cancer cell lines: HCC827 and PC9 (eight samples each). Each cell line includes a wild-type control designated as “parental” and three distinct Gefitinib-resistant derivatives (GR) obtained through 6-month treatment protocols: continuous exposure with incrementally increasing Gefitinib concentrations (GR-High), constant low-dose exposure (GR-Low) and intermittent exposure (GR-Pulse).

**Analysis performed:** we performed 12 different default complete RNA-seq analyses in the *Perform a Complete Analysis* section:

1. *HCC827-GR-High vs. HCC827-parental, UP-genes*
2. *HCC827-GR-Low vs. HCC827-parental, UP-genes*
3. *HCC827-GR-Pulse vs. HCC827-parental, UP-genes*
4. *HCC827-GR-High vs. HCC827-parental, DOWN-genes*
5. *HCC827-GR-Low vs. HCC827-parental, DOWN-genes*
6. *HCC827-GR-Pulse vs. HCC827-parental, DOWN-genes*
7. *PC9-GR-High vs. PC9-parental, UP-genes*
8. *PC9-GR-Low vs. PC9-parental, UP-genes*
9. *PC9-GR-Pulse vs. PC9-parental, UP-genes*
10. *PC9-GR-High vs. PC9-parental, DOWN-genes*
11. *PC9-GR-Low vs. PC9-parental, DOWN-genes*
12. *PC9-GR-Pulse vs. PC9-parental, DOWN-genes*

Subsequently, these 12 result files were subjected to pathway enrichment analysis using the *Enrichment Analysis and Result Interpretation* module. Comprehensive results and visualizations for all 12 analyses listed above are provided at https://github.com/franruss/REDAC/tree/main/usage%20example, while the user manual, in which all these analyses are described, is provided at https://github.com/franruss/REDAC/blob/main/docs/REDAC_user_manual.pdf.

In this section, the tabs *A possible interpretation (by Gemma)* and *Another possible interpretation (by LLaMA)* were used to obtain expert-level assistance in the biological interpretation of the enrichment results. In this use case, we present detailed results exclusively for the comparison of *HCC827-GR-Pulse vs. HCC827-parental UP-regulated genes*. Similar analyses were performed for the remaining 11 comparisons listed above, and the same results reported in (Frezzetti *et al.* 2024) were obtained in a straightforward manner.

**Summary of the advantages of using REDAC:** 

- *Immediate and interpretable results*: REDAC identified 1291 up regulated genes, including key markers previously reported in (Frezzetti *et al.* 2024), such as: ALDH1A1, ALDH1A3, ALDH1L2, ABCB1, FZD4, FZD1, FZD2, FDZ7, and CD44.
- *Biologically relevant KEGG pathways*: Key pathways identified as up regulated in GR pulse cell lines include TNF signaling, MAPK signaling, IL-17 signaling, NF-kappa B signaling, RAS signaling, epithelial cell signaling, Fc gamma R-mediated phagocytosis, apoptosis, and regulation of TRP channels inflammatory mediators.
- *Intuitive visualization*: Dot plots provide clear and immediate graph for the representation of those enriched pathways.
- *Network visualization*: KEGG network plots illustrate enriched pathway interconnections and their associated genes.
- *Expert-level interpretation through dual LLM analysis*: To enhance the contextual accuracy of the LLM-generated interpretations, we provided a descriptive request that specifies the experimental context and biological question. This contextual information enabled the LLMs to generate more precise and relevant interpretations of the enrichment results. For this comparison, we queried both models with: *“*Explain these results produced by the comparison of pulse induced drug resistance in HCC827 cells against their parental cells to study the mechanisms induced by this suboptimal dose of Gefitinib.” This description is important because REDAC uses it to extract keywords (such as: “HCC827,” “drug resistance,” “Gefitinib”) that guide the retrieval of relevant articles from PubMed to build the RAG module. Both Gemma and LLaMA provided a comprehensive summary of results with detailed interpretations based on the RAG module.
- *Clear and comprehensive result presentation*: Results are displayed through complete and informative visualizations.
- *Enhanced reproducibility*: Complete and transparent reports are automatically generated in dual formats—static Word documents and interactive HTML reports—with a single click. The Word report serves as suitable Supplementary Material, available as supplementary data at *Bioinformatics Advances* online, for publications. The HTML report features fully interactive plots where users can zoom in, explore data points, and access detailed information including gene names, sample identifiers, and quantitative values.

## 5 Quantitative analysis of hallucination reduction and discussion

As previously described, REDAC employs the JSON format as an intermediary layer to execute analyses. In this framework, LLMs are restricted from generating new R code and can instead produce only JSON structures with predefined keys. This choice ensures that the models supply only the parameters required to execute a set of predefined RNA-seq analysis functions, which have been written and fixed in advance. Consequently, LLMs cannot modify or create new analytical code, thereby preventing code-level hallucinations. Their role is limited to assisting users in selecting the appropriate parameters for the desired analysis. Each JSON key corresponds to a predefined R function (e.g. rnaseq_analysis(), pathway_enrichment(), etc.). As a result, the generation of syntactically invalid or functionally unverified code is structurally impossible. While the JSON constraint effectively prevents syntactic hallucinations and arbitrary code execution, it does not fully eliminate potential semantic misinterpretations (e.g. a mis-specified comparison). To assess the impact of JSON constraints on hallucination reduction, we conducted 150 tests using 15 natural language prompts in 10 RNA-seq datasets obtained from www.cbioportal.org and www.ncbi.nlm.nih.gov/geo. Therefore, we ran 150 tests in the JSON-restricted mode and 150 tests in the free-text mode (i.e., without applying any JSON restrictions). The results, summarized in Table 3, show that 75.3% of the tests were successful, while 24.7% failed. In this analysis, we categorized the most common types of hallucinations as follows:

**Table 3.** Comparison of hallucination frequency between Free-text and JSON-constrained modes in REDAC (all details and results of tests are available at https://github.com/franruss/REDAC/tree/main/validation).

Failure type	Free-text (%)	JSON (%)
Successful tests	11.0	75.3
Failed tests (due to hallucinations)^1^	89.0	24.7
1-Parsing errors	0	23.3
2-Unrecognized functions	0	0
3-Conceptual errors	30.0	0
4-Execution errors	59.0	1.4
5-Invalid sample references	0	0

1 In this table, we evaluated the contribution of each type of hallucination to the failure of tests and their frequencies in both the **Free-text** requests and the **JSON** constrained requests to REDAC.

1. *Parsing errors—*caused by unrecognized or ambiguous terms in the user-provided description.
2. *Unrecognized functions—*occurred when the generated code references functions that are undefined or unavailable, preventing execution.
3. *Conceptual errors—*occurred when the code runs without technical issues, but the resulting output is biologically or analytically incorrect.
4. *Execution errors—*arisen from syntactic mistakes in the generated code or from temporary connection issues with the LLM APIs.
5. *Invalid sample references—*occurred when the requested samples were not present in the data or when the model incorrectly interprets sample identifiers.

Among the failed tests, 23.3% were due to *Parsing errors*, primarily related to misinterpretations of the words “de,” “up” and “down” that are crucial to understand which type of regulated genes the user asked (“de” stands for: “differentially expressed”, “up”: “up regulated” and “down” for “down regulated” genes). In this particular case, rather than leaving the user with this error, we adopted the workaround of running the RNA-seq analysis anyway to generate differentially expressed genes. Therefore, in real use, the 23.3% reported in Table 3 has been reduced. Notably, no *Conceptual errors* occurred in any of the JSON tests, confirming that the JSON constraint effectively prevents the LLM from producing or executing unverified code. The adoption of JSON constraints eliminated all *Conceptual errors* and the *Execution errors* dropped to 1.4% due to API connection errors only. In general, the hallucination rate decreased from a total of 89% to 24.7%. In this comparison, we evaluated a version of REDAC without JSON restrictions (available at: https://frusso.shinyapps.io/REDAC_NO_JSON), where the LLM (Llama) freely generated blocks of R code. We saved and executed these code blocks to test their functionality. In this unrestricted mode, Llama frequently produced incorrect design matrices, leading to invalid comparisons and nonsensical results. Specifically, the LLM often generated the incorrect code line: model.matrix(∼0+groupsubset) instead of the correct model.matrix(∼1+groupsubset)), resulting in that every gene in the user input data being significantly differentially expressed regardless of its actual expression. We classified this as *Conceptual errors* in Table 3. Only when explicitly prompted to “use the DESeq package” did the LLM generate two alternative codes: the first using *glmLRT()* (still with an incorrect model) and the second using the *DESeq()* function. The latter produced correct results (representing 11.0% of the successful tests in Table 3).

The findings reported in Table 3 highlight a critical risk in relying on a free-text (i.e. unconstrained) code generated by an LLM: even when syntactically correct, it might encode conceptual flaws that produce scientifically invalid results. Detecting such errors requires both statistical expertise and deep programming skills that many users of RNA-seq tools may lack. By contrast, REDAC enables users without prior training in statistics or programming to perform analyses in a correct, transparent and reproducible way. All analyses are fully documented and detailed reports are automatically generated, including the R code used, ensuring reproducibility and verifiability. Running free-text LLM code, by comparison, risks either execution failures (requiring coding intervention) or, worse, apparently valid results based on incorrect analytical logic. For more details, all test results, datasets, prompts, generated code, and validation scripts are publicly available at https://github.com/franruss/REDAC/tree/main/validation.

## 6 Conclusions and future developments

REDAC advances RNA-seq analysis democratization by addressing critical limitations in existing bioinformatics chatbots. Unlike BioMANIA, and BIA, which occasionally suffer from hallucinations, limited language support, and poor code transparency, REDAC integrates standardized pipelines with dual LLM interpretation. Key innovations include hallucination management through structured JSON formatting, transparent code generation with expert-level alternatives, multiple biological perspectives through dual LLM integration, and comprehensive workflow coverage from raw counts to pathway enrichment. The Gefitinib resistance case study identified relevant EMT, p53, and TNF-alpha signaling pathways, demonstrating the practical utility of the research. REDAC’s R implementation leverages established packages like edgeR, offers superior statistical modeling, and provides both static and interactive outputs for reproducible research.

Future developments will leverage advanced foundation models fine-tuned for biological data interpretation and incorporate retrieval-augmented generation for real-time literature integration to keep pace with rapidly evolving genomics knowledge. Key expansion areas include the integration of single cell RNA-seq analysis (Vandereyken *et al.* 2023) with workflows such as Seurat and Scanpy to address the field’s growth and unique analytical demands and the addition of support for spatial transcriptomics using Seurat’s spatial modules for spatially resolved gene expression analysis (Rich et al. 2024).

Multi-omics integration will enable the concurrent analysis of transcriptomics, proteomics, and epigenomics data through established frameworks (De Filippis *et al.* 2025). Enhanced statistical modeling will accommodate complex experimental designs, such as time-series and Bayesian methods for uncertainty quantification. Improvements in natural language processing will facilitate better query understanding through conversational interfaces, while automated benchmarking will regularly validate analytical results against reference datasets, maintaining accuracy and reliability as the platform expands to meet current and future genomics research needs from basic science to clinical applications.

## Supplementary Material

vbaf321_Supplementary_Data

## Data Availability

The code and data are available at https://github.com/franruss/REDAC ahttps://github.com/franruss/REDAC/blob/main/docs/REDAC_user_manual.pdf. The input file used in the case study is named “Raw data RNA-seq for submission with gene name 3 1 24.xlsx” and is available at https://zenodo.org/records/11057181. The results and visualizations for the 12 analyses reported in the case study are provided at https://github.com/franruss/REDAC/tree/main/usage%20example. All test results, datasets, prompts, generated code, and validation scripts are publicly available at https://github.com/franruss/REDAC/tree/main/validation.
