# Supplementary material for: REDAC: RNA-seq expression data analysis chatbot
Source: Bioinform Adv. 2025 Dec 27;6(1):vbaf321. doi: 10.1093/bioadv/vbaf321 (PMC12927421; doi:10.1093/bioadv/vbaf321)
Supplement: vbaf321_Supplementary_Data [file vbaf321_supplementary_data.pdf]

REDAC

User Manual*

Giovanni Maria De Filippis[1] and Francesco Russo[2]

[1]Department of Electrical Engineering and Information Technology,
University of Naples Federico II, Naples, Italy

[2]Consiglio Nazionale delle Ricerche, IEOMI, Naples, Italy

November 2025

Contents

1 Introduction **3**
1.1 Local Installation . . . 3
1.1.1 Prerequisites . . . 3
1.1.2 Install required R packages . . . 3
1.1.3 Install Bioconductor packages . . . 3
1.1.4 Run REDAC locally . . . 4
1.2 REDAC Architecture . . . 4

2 How to use the Perform a Complete Analysis module **4**

3 How to use the Enrichment Analysis and Result Interpretation module **15**

4 How to use the Plot Generation module **20**

***Acknowledgements**: we are thankful to
1. Ministero dell'Università e della Ricerca (**MUR**), Italy,
2. Department of Electrical Engineering and Information Technology, University of Naples Federico II, Naples, Italy,
3. IRCCS Istituto Romagnolo per lo studio dei Tumori "Dino Amadori" (IRST), Meldola (FC), Italy,
4. Cell Biology and Biotherapy Unit, Istituto Nazionale Tumori-IRCCS-Fondazione G. Pascale, Naples, Italy.

Funding: this work has been funded by the **MUR (PRIN2022PNRR-2022MZJR9X, P2022YA3LL)**.

to Luisa

1 Introduction

This user manual introduces REDAC, a web application that features a graphical user interface for the analysis of bulk RNA-Seq count data (non-negative integers). The application is developed in the R programming language (http://cran.r-project.org/), an open-source, object-oriented environment for statistical computing and graphics. REDAC integrates several RNA-Seq analysis tools available through Bioconductor (www.bioconductor.org).

Availability: REDAC is an open source software released under the GPL3 license.

Available for local use at: https://github.com/franruss/REDAC

Available for online use at: https://frusso.shinyapps.io/REDAC

Contact: francesco.russo@cnr.it

1.1 Local Installation

As a shiny app, REDAC does not need an installation. If you want to use REDAC locally, you just need to download the code from https://github.com/franruss/REDAC.

1.1.1 Prerequisites

1. R (version >= 4.5.0),
2. RStudio (recommended),
3. Internet connection for package installation.

1.1.2 Install required R packages

Please, install all the required CRAN packages by running the code below in your R environment:

```
install.packages(c("shiny","shinydashboard","shinythemes","ggplot2","plotly",

"gplots","lattice","mgcv","vioplot","httr","jsonlite","stringr","knitr","tidyr",

"dplyr","igraph","FactoMineR","factoextra","plot3D","ggraph","ggrepel","markdown",

"msigdbr", "text2vec", "rentrez"))
```

1.1.3 Install Bioconductor packages

Please, install all the required Bioconductor packages packages by running the code below:

```
if (!require("BiocManager", quietly = TRUE))

install.packages("BiocManager")

BiocManager::install(c("SummarizedExperiment","edgeR","preprocessCore", "ReactomePA",

"AnnotationDbi","clusterProfiler","org.Hs.eg.db","enrichplot","DOSE","rWikiPathways",

"reactome.db", "GO.db"))
```

1.1.4 Run REDAC locally

Now, you have to create an *.Renviron* file template if it does not exist. In this file, you have to write just a line of code in which you pass your personal API key to the variable *API_KEY*. This variable is read by the REDAC *serve.R* code and is used to interrogate the AI models at https://www.together.ai. So, in *.Renviron* add this line and provide your personal API key in the following way:

```
API_KEY = "add_your_api_key_here"
```

If you do not possess a personal API key, you need to create one at https://www.together.ai and add it to the *.Renviron* file as described before.

Finally, run the following line of code in an R environment:

```
shiny::runApp()
```

And the REDAC main page will appear on your screen ready to use.

Alternatively, you can follow the step by step instructions described in the file "setup.R" available here: https://github.com/franruss/REDAC/blob/main/setup.R to install all required packages straightforwardly.

1.2 REDAC Architecture

The main interface of REDAC is organized into three modules, each corresponding to a key step in the data analysis workflow.

1. **Perform a Complete Analysis**: executes the entire RNA-Seq analysis pipeline from start to finish.
2. **Enrichment Analysis and Result Interpretation**: focuses on the interpretation of enrichment results derived from differential expression analysis.
3. **Plot Generation**: allows users to generate individual plots independently of the full analysis workflow.

2 How to use the Perform a Complete Analysis module

The user interface (shown in Figure 1) constitutes the *Perform a Complete Analysis* module. This module guides the user through a complete bulk RNA-seq differential expression data analysis using raw count data as input via natural language interaction with the Gemma LLM.

To initiate the analysis workflow, users begin by clicking the "Browse" button to upload a tab-separated values (TSV) file containing the RNA-Seq count matrix (Figure 2). The input file format requires genes as rows and samples as columns, with raw count values representing expression levels.

Upon successful file upload, the data contents are immediately displayed for preliminary inspection. The interface provides flexible viewing options, allowing users to examine the first 10, 50, or 100 rows of the data set. A search functionality enables rapid gene-specific queries by entering gene identifiers in the designated search box (Figure 2).

After data upload, REDAC automatically generates a comprehensive suite of diagnostic visualizations (Figures 3 and 4) to facilitate quality assessment and identification of potential data anomalies, including sample mislabeling or aberrant count distributions. Users can navigate between different visualization types through the tabbed interface, as demonstrated in Figure 4 where the three-dimensional PCA plot is selected.

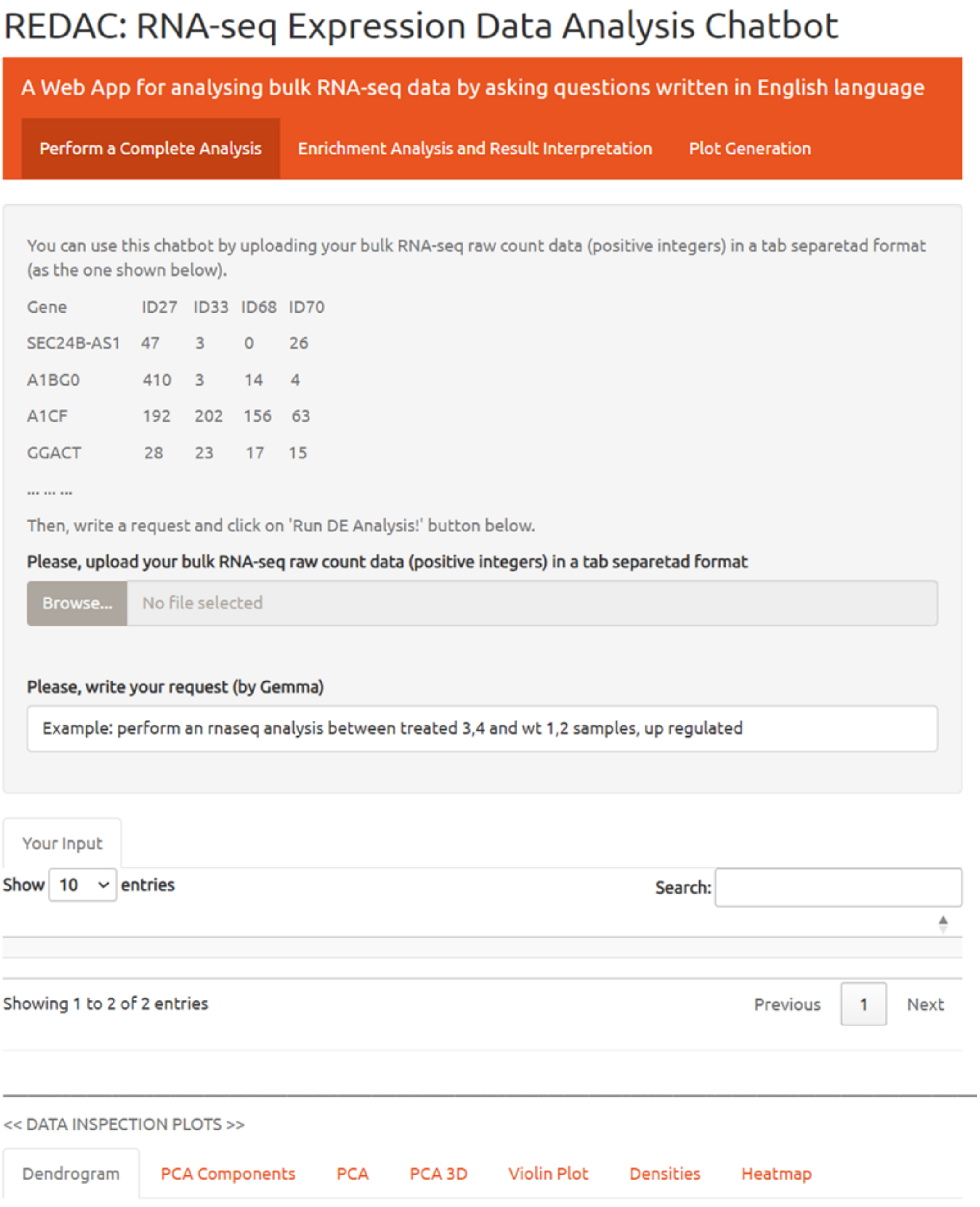

Figure 1: REDAC main interface.

The analysis is initiated through a natural language interface where users specify their experimental comparison of interest. This includes defining the sample groups for statistical testing

using edgeR methodology and specifying the direction of differential expression analysis (up regulated, down regulated, or de regulated). For instance, a query might be: “execute an rnaseq analysis between treated 3,4 and wt 1,2 samples, de regulated” as the one shown in Figure 2 in red circle.

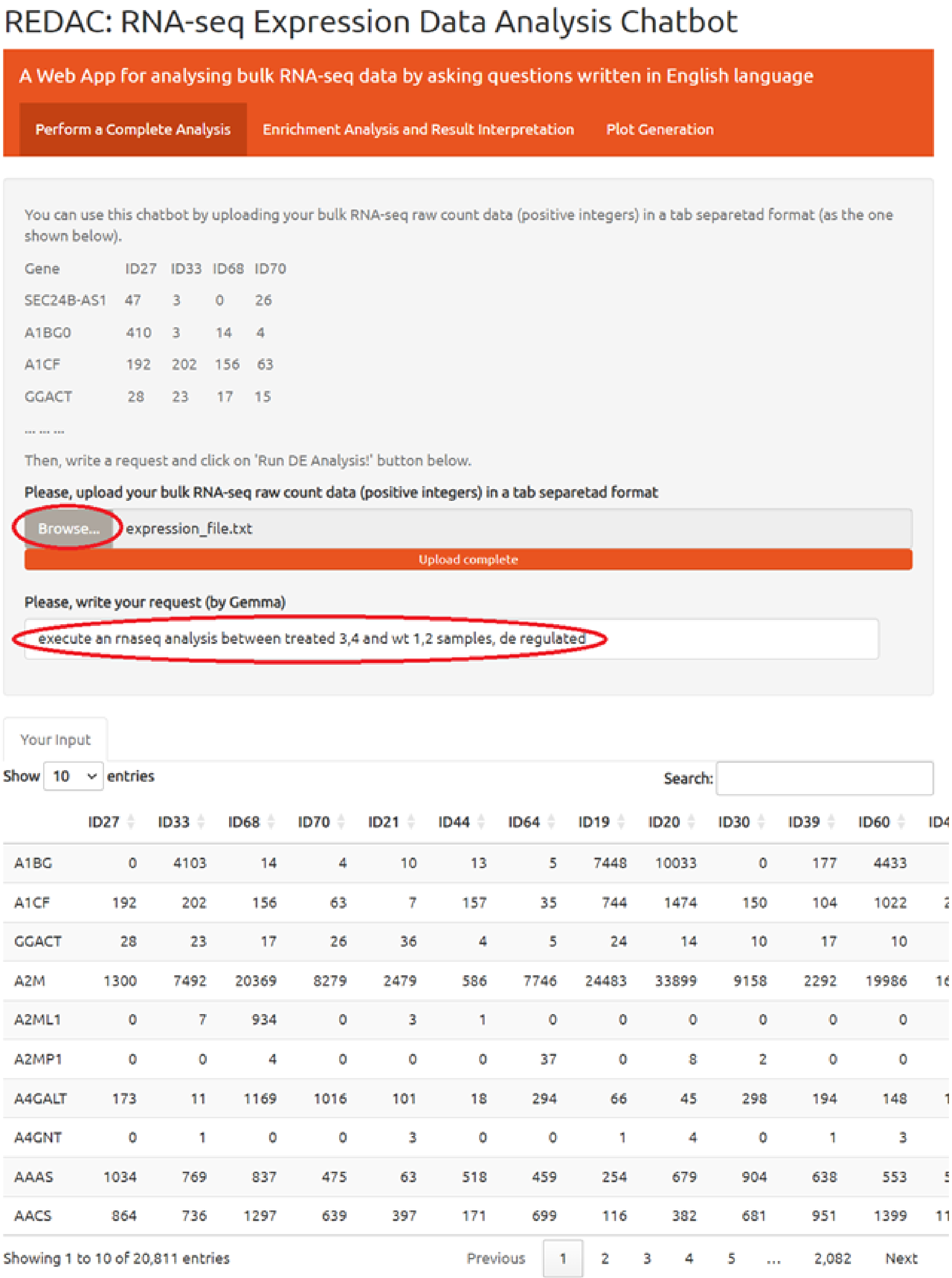

Figure 2: File upload interface showing the Browse button (highlighted in red circle) for uploading tab-separated value files containing RNA-Seq count data. Users must specify their analytical request by defining sample comparisons for edgeR differential expression testing and indicating the direction of interest (up regulated, down regulated, or de regulated).

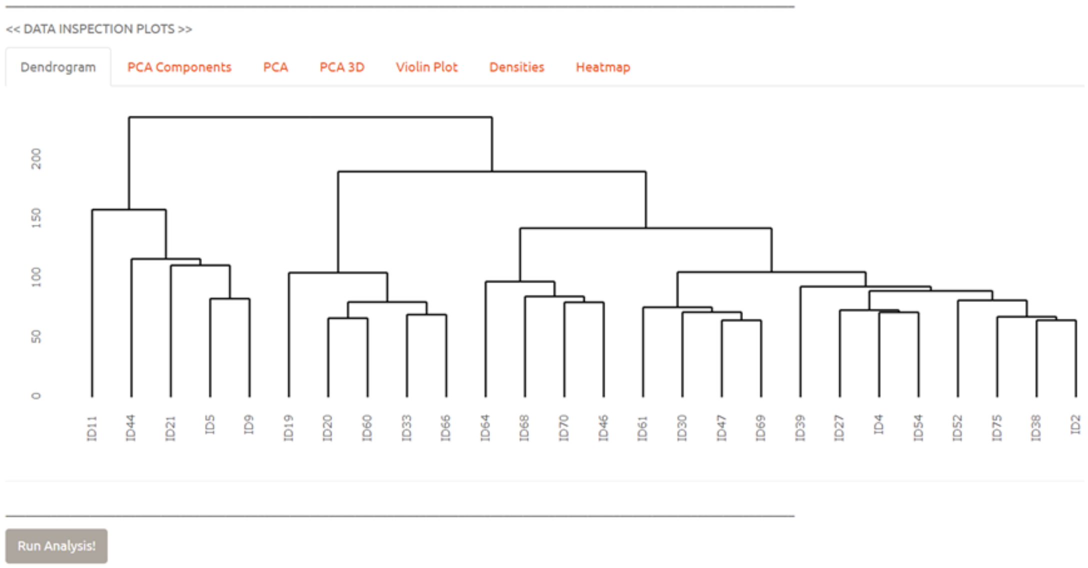

Figure 3: Automated quality assessment visualizations generated following gene expression file upload. These diagnostic plots enable users to identify potential data anomalies, including sample mislabeling or aberrant count distributions. The dendrogram shown here illustrates sample clustering based on expression profiles.

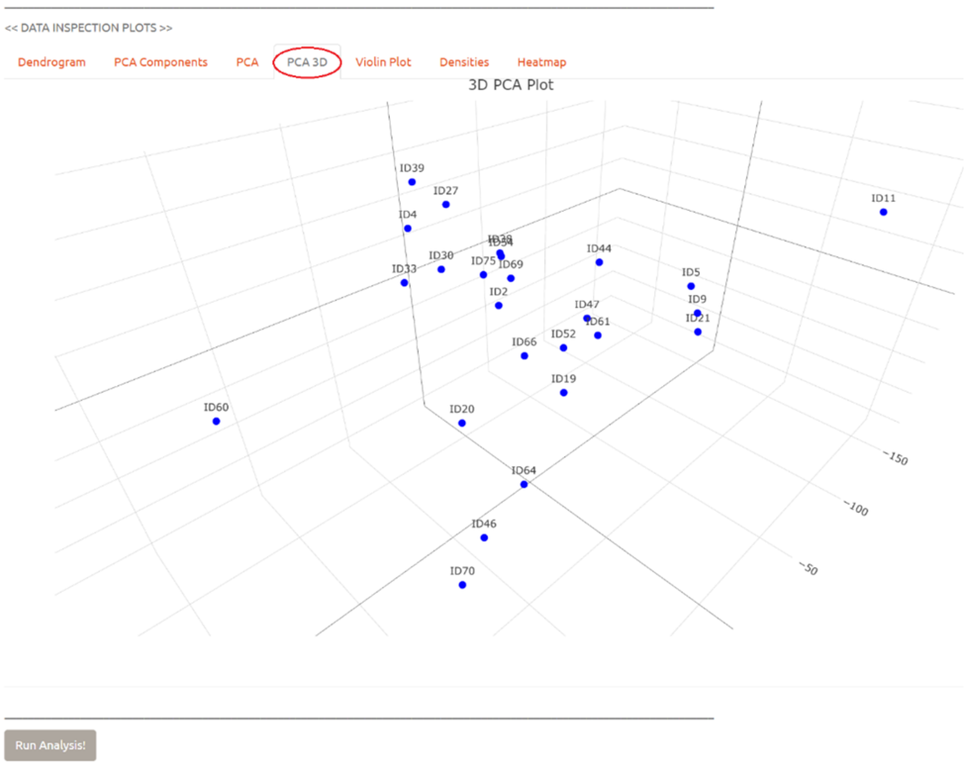

Figure 4: Interactive visualization interface demonstrating navigation between different plot types through tabbed selection. The three-dimensional principal component analysis (PCA 3D) tab is shown selected (highlighted in red circle).

Once the data have been inspected and the request has been written, we can click the Run

Analysis! button. Subsequently, a yellow pop-up appears on the screen with a waiting message and a progress bar is shown (see Figure 5).

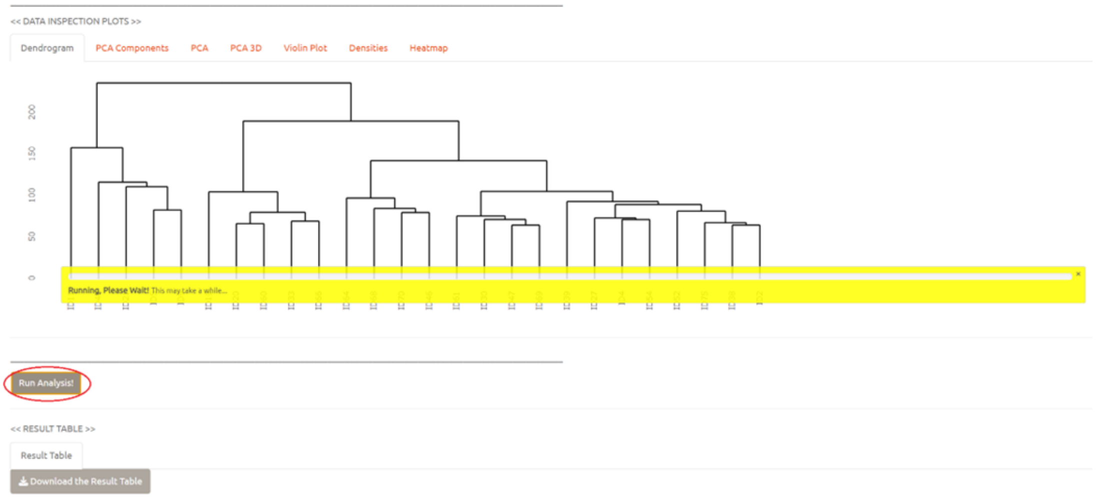

Figure 5: Analysis execution interface showing the progress notification system activated upon selecting the Run Analysis! button. The yellow notification popup provides real-time status updates with an integrated progress bar.

When the execution is completed, the first 10 lines of results are shown. We can download the entire result table by clicking the Download the Result Table button (see Figure 6).

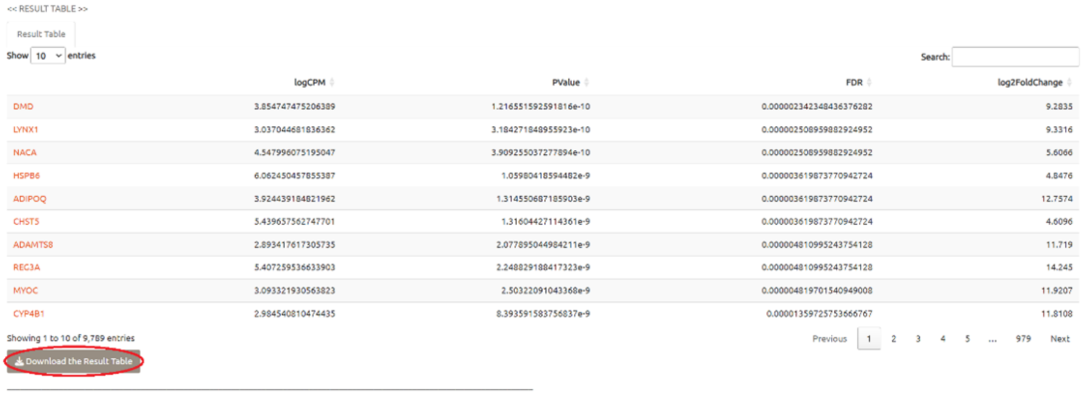

<< RESULT TABLE >>

Result Table

Show 10 entries

Search:

	logCPM	PValue	FDR	log2FoldChange
DMD	3.854747475206389	1.216551592591816e-10	0.000002342348436376282	9.2835
LYNX1	3.037044681836362	3.184271848955923e-10	0.000002508959882924952	9.3316
NACA	4.547996075195047	3.909255037277894e-10	0.000002508959882924952	5.6066
HSPB6	6.062450457855387	1.05980418594482e-9	0.000003619873770942724	4.8476
ADIPOQ	3.924439184821962	1.314550687185903e-9	0.000003619873770942724	12.7574
CHST5	5.439657562747701	1.31604427114361e-9	0.000003619873770942724	4.6096
ADAMTS8	2.893417617305735	2.077895044984211e-9	0.000004810995243754128	11.719
REG3A	5.407259536633903	2.248829188417323e-9	0.000004810995243754128	14.245
MYOC	3.093321930563823	2.50322091043368e-9	0.000004819701540949008	11.9207
CYP4B1	2.984540810474435	8.393591583756837e-9	0.0000135972575366767	11.8108

Showing 1 to 10 of 9,789 entries

Previous 1 2 3 4 5 ... 979 Next

Download the Result Table

Figure 6: Results summary interface displaying the top 10 differentially expressed genes upon analysis completion. The complete results table can be downloaded using the designated button (highlighted in red circle).

Moreover, a Volcano Plot and a MA Plot are automatically generated to evaluate results globally (see Figure 7). If we put the mouse pointer on a red dot, the corresponding gene name will appear on the screen (see Figure 7).

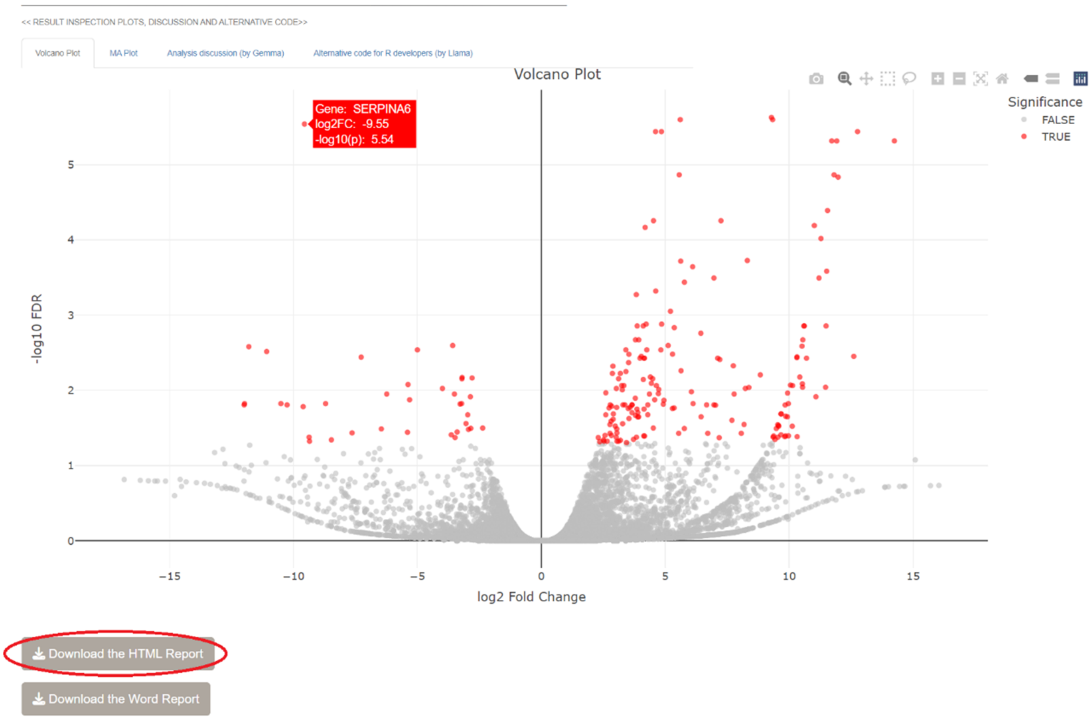

Figure 7: Automated generation of volcano plot and MA plot visualizations for the evaluation of differential expression results. Interactive functionality allows gene identification through mouse-over display of gene names.

At this stage, REDAC provides multiple pathways for result interpretation and further analysis tailored to different user expertise levels. Users new to RNA-seq analysis can access the “Analysis Discussion (by Gemma)” feature to obtain a guided explanation of the results (see Figure 8). This LLM provides comprehensive descriptions of the typical analytical steps in RNA-seq workflows and recommends appropriate methodological approaches for each stage.

For experienced R users seeking greater analytical flexibility, the “Alternative code for R developers (by LLaMA)” option generates customized R scripts that implement the same analysis using alternative functions or parameter configurations (see Figure 9). This feature enables advanced users to explore different analytical approaches while maintaining reproducibility.

Additionally, users can generate a comprehensive, interactive HTML report by selecting “Download the HTML Report” (see Figure 7). This report provides complete analytical documentation including detailed descriptions of each analytical step, all generated visualizations, and the complete R code executed during the analysis. The HTML format ensures cross-platform compatibility and enables interactive exploration of results in any standard web browser (see Figures 10, 11, and 12).

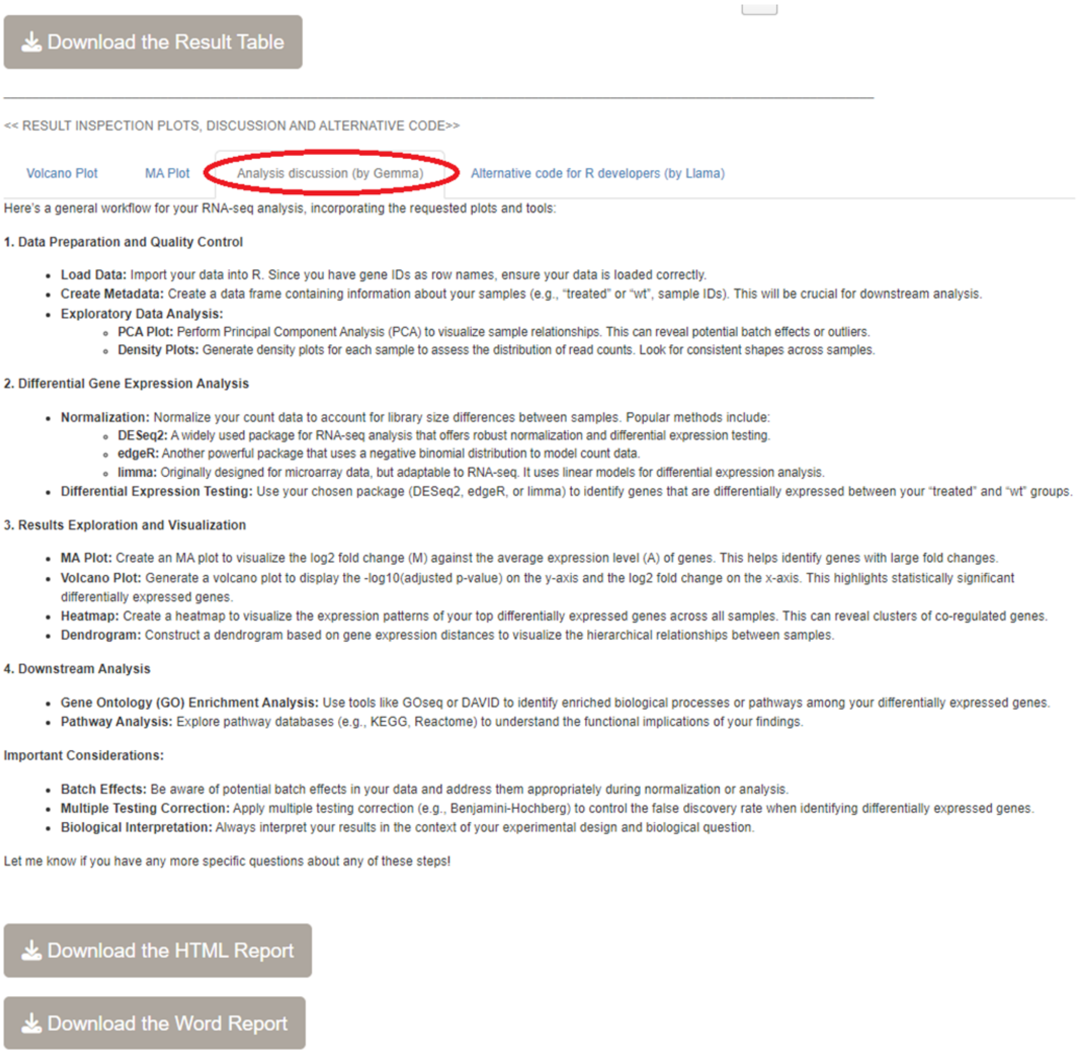

Figure 8: Analysis Discussion interface powered by Gemma LLM. This feature provides comprehensive explanations of RNA-seq analysis workflows, detailing the standard analytical steps and recommended methodological approaches for each stage.

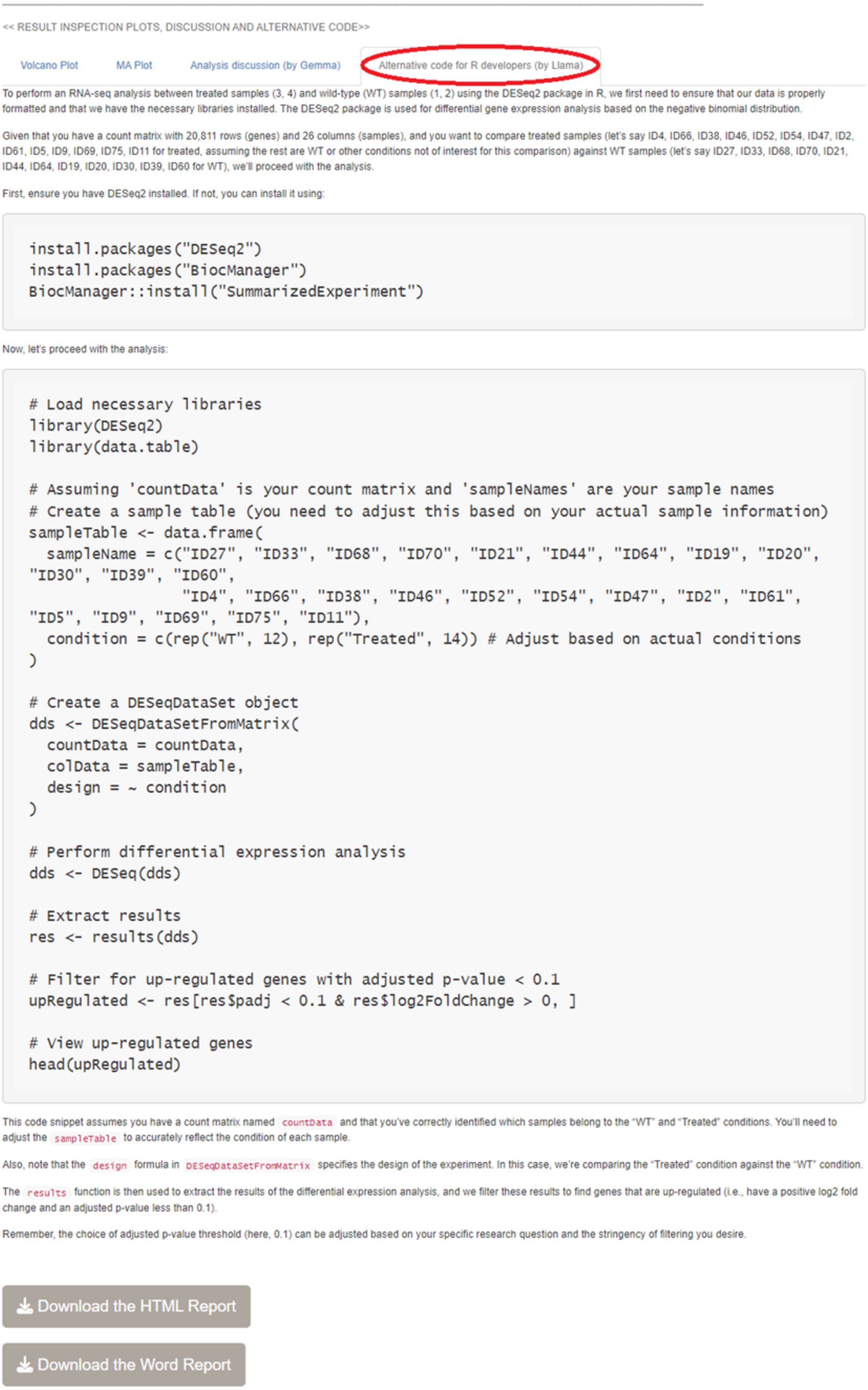

<< RESULT INSPECTION PLOTS, DISCUSSION AND ALTERNATIVE CODE>>

Volcano Plot | MA Plot | Analysis discussion (by Gemma) | Alternative code for R developers (by Llama)

To perform an RNA-seq analysis between treated samples (3, 4) and wild-type (WT) samples (1, 2) using the DESeq2 package in R, we first need to ensure that our data is properly formatted and that we have the necessary libraries installed. The DESeq2 package is used for differential gene expression analysis based on the negative binomial distribution.

Given that you have a count matrix with 20,811 rows (genes) and 26 columns (samples), and you want to compare treated samples (let's say ID4, ID66, ID38, ID46, ID52, ID54, ID47, ID2, ID61, ID5, ID9, ID69, ID75, ID11 for treated, assuming the rest are WT or other conditions not of interest for this comparison) against WT samples (let's say ID27, ID33, ID68, ID70, ID21, ID44, ID64, ID19, ID20, ID30, ID39, ID60 for WT), we'll proceed with the analysis.

First, ensure you have DESeq2 installed. If not, you can install it using:

```
install.packages("DESeq2")
install.packages("BiocManager")
BiocManager::install("SummarizedExperiment")
```

Now, let's proceed with the analysis:

```
# Load necessary libraries
library(DESeq2)
library(data.table)

# Assuming 'countData' is your count matrix and 'sampleNames' are your sample names
# Create a sample table (you need to adjust this based on your actual sample information)
sampleTable <- data.frame(
  sampleName = c("ID27", "ID33", "ID68", "ID70", "ID21", "ID44", "ID64", "ID19", "ID20",
"ID30", "ID39", "ID60",
                 "ID4", "ID66", "ID38", "ID46", "ID52", "ID54", "ID47", "ID2", "ID61",
"ID5", "ID9", "ID69", "ID75", "ID11"),
  condition = c(rep("WT", 12), rep("Treated", 14)) # Adjust based on actual conditions
)

# Create a DESeqDataSet object
dds <- DESeqDataSetFromMatrix(
  countData = countData,
  colData = sampleTable,
  design = ~ condition
)

# Perform differential expression analysis
dds <- DESeq(dds)

# Extract results
res <- results(dds)

# Filter for up-regulated genes with adjusted p-value < 0.1
upRegulated <- res[res$padj < 0.1 & res$log2FoldChange > 0, ]

# View up-regulated genes
head(upRegulated)
```

This code snippet assumes you have a count matrix named `countData` and that you've correctly identified which samples belong to the "WT" and "Treated" conditions. You'll need to adjust the `sampleTable` to accurately reflect the condition of each sample.

Also, note that the `design` formula in `DESeqDataSetFromMatrix` specifies the design of the experiment. In this case, we're comparing the "Treated" condition against the "WT" condition.

The `results` function is then used to extract the results of the differential expression analysis, and we filter these results to find genes that are up-regulated (i.e., have a positive log2 fold change and an adjusted p-value less than 0.1).

Remember, the choice of adjusted p-value threshold (here, 0.1) can be adjusted based on your specific research question and the stringency of filtering you desire.

Download the HTML Report

Download the Word Report

Figure 9: Alternative R code generation interface powered by LLaMA. This feature generates customized R scripts implementing equivalent analyses using alternative functions or parameter configurations, enabling experienced users to explore different analytical approaches.

Report of the Analysis

Design and Dispersion

```
Sys.time()
```

```
## [1] "2025-07-31 16:12:03 CEST"
```

```
counts <- params$counts
res <- params$res
#treated_samples <- colnames(counts)[as.numeric(params$treated_samples)]
#wt_samples <-      colnames(counts)[as.numeric(params$wt_samples)]
#which_results <- params$which_results

# Build metadata
#treated_condition <- data.frame(patients = treated_samples, condition = 'treated')
#wt_condition <- data.frame(patients = wt_samples, condition = 'wt')
#samples <- rbind(wt_condition, treated_condition)
#rownames(samples) <- samples$patients

# Subset and order count matrix
#dataset <- counts[, samples$patients]
#samples$condition <- factor(samples$condition, levels = c('wt', 'treated'))

# Create DGEList
#dge <- DGEList(counts = dataset, group = samples$condition)

# Filter low counts
#keep <- rowSums(counts) >= dim(counts)[2]
#dge <- dge[keep, , keep.lib.sizes = FALSE]

# Normalize and estimate dispersion
#dge <- calcNormFactors(dge)
#dge <- estimateDisp(dge)

# Differential expression
#et <- exactTest(dge, pair = c('wt', 'treated'))
#res <- topTags(et, n = nrow(dge))$table
#res <- res[order(res$FDR), ]
#res$log2FoldChange <- round(res$logFC, 4)
#res$logFC <- NULL

# Filter DE results
#if (which_results == 'down') {
#  DEres <- res[res$log2FoldChange < 0, ]
#} else if (which_results == 'up') {
#  DEres <- res[res$log2FoldChange > 0, ]
#} else {
#  DEres <- res
#}
#DEres <- na.omit(DEres)

# Plot
#plotBCV(dge)
```

Top 100 Differentially Expressed Genes

```
knitr::kable(head(res, 100), caption = 'Top 100 Differentially Expressed Genes')
```

Top 100 Differentially Expressed Genes

	logCPM	PValue	FDR	log2FoldChange

Figure 10: Header section of the automatically generated HTML report, providing comprehensive analytical documentation.

```
#plotBCV(dge)
```

Top 100 Differentially Expressed Genes

```
knitr::kable(head(res, 100), caption = 'Top 100 Differentially Expressed Genes')
```

Top 100 Differentially Expressed Genes

	logCPM	PValue	FDR	log2FoldChange
DMD	3.854748	0.00e+00	0.0000023	9.2835
LYNX1	3.037045	0.00e+00	0.0000025	9.3316
NACA	4.547996	0.00e+00	0.0000025	5.6066
HSPB6	6.062450	0.00e+00	0.0000036	4.8476
ADIPOQ	3.924439	0.00e+00	0.0000036	12.7574
CHST5	5.439658	0.00e+00	0.0000036	4.6096
ADAMTS8	2.893418	0.00e+00	0.0000048	11.7190
REG3A	5.407260	0.00e+00	0.0000048	14.2450

Figure 11: Top 100 differentially expressed genes as displayed in the interactive HTML report, showing detailed statistical results and gene annotations.

```r
# Plot Dendrogram
x = counts
x = log10(x+1)
colnames(x) <- colnames(counts)
rownames(x) <- rownames(counts)
clustering_used_method <- 'ward.D'
distance_used_method <- 'euclidean'
hclustfunc <- function(x) hclust(x, method = clustering_used_method)
distfunc <- function(x) dist(x, method = distance_used_method)
fit <- hclustfunc(distfunc(t(x)))
# Convert hclust to dendrogram and then to dendrogram segments
dend <- as.dendrogram(fit)
p <- ggdendrogram(dend , rotate = FALSE, size = 2)
ggplotly(p)
```

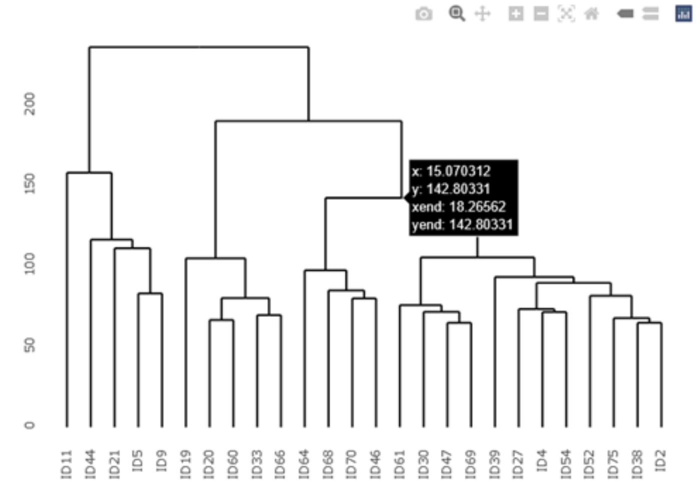


```r
# Principal Component Histogram
# Step 1: Remove genes (rows) with zero variance
counts <-  counts[apply( counts, 1, var) != 0, ]
pca_result <- prcomp(t(counts), scale. = TRUE)  # Transpose if samples are columns
# Calculate variance explained
var_explained <- pca_result$sdev^2 / sum(pca_result$sdev^2) * 100
# Create Plotly screeplot
scree_plot <- plot_ly(
  x = paste0('PC', 1:length(var_explained)),
  y = var_explained,
  type = 'bar',
  marker = list(color = 'blue'),
  text = ~paste0(round(var_explained, 2), '%'),
  hoverinfo = 'text'
) %>%
  layout(
    title = 'Principal Component Histogram',
    xaxis = list(title = 'Principal Components'),
    yaxis = list(title = 'Variance Explained (%)')
  )
scree_plot
```

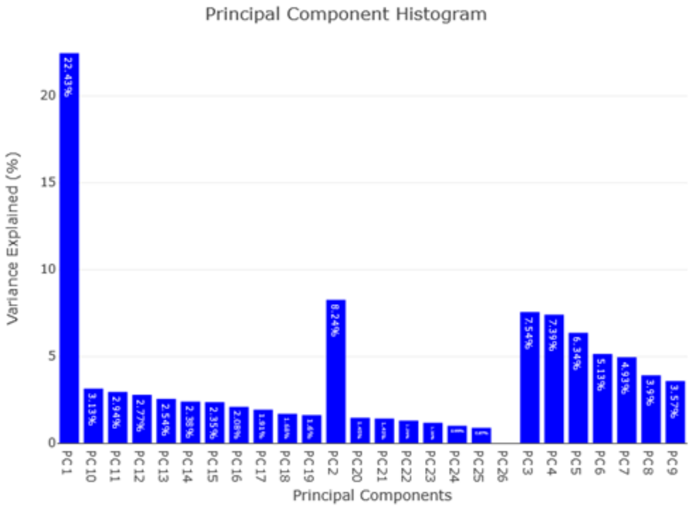


```r
# Principal Component Analysis
x <- counts
# Step 1: Remove genes (rows) with zero variance
x <- x[apply(x, 1, var) != 0, ]
```

Figure 12: Representative section of the HTML report demonstrating the analytical documentation format.

Upon downloading the differential expression results table, users can navigate to the second tab, “Enrichment Analysis and Result Interpretation”, to upload this file for subsequent pathway enrichment analysis.

3 How to use the Enrichment Analysis and Result Interpretation module

This module facilitates biological interpretation of differential expression results through pathway enrichment analysis. Users initiate the process by clicking the “Browse” button to upload an edgeR result file, as demonstrated in Figure 13. Following successful file upload, the interface displays the first 10 lines of the input data for verification purposes.

To enhance the contextual accuracy of the LLM-generated interpretations, users should provide a descriptive request that specifies the experimental context and biological question. This contextual information enables the LLMs to generate more precise and relevant interpretations of the enrichment results. For example, a comprehensive request might state: *“Explain these results produced from the comparison of pulse-induced drug resistance in HCC827 cells against their parental cells to study the mechanisms induced by this suboptimal dosage of Gefitinib”* (Figure 13). After entering the contextual description, users initiate the analysis by clicking the “Enrich!” button.

The system then performs KEGG pathway enrichment analysis on the uploaded differential expression data. Upon completion, the enrichment results are displayed in a tabular format (Figure 14). Users can visualize the most significantly enriched KEGG pathways by selecting the “Dot Plot” tab (Figure 15), which generates a comprehensive dot plot visualization displaying pathway enrichment statistics.

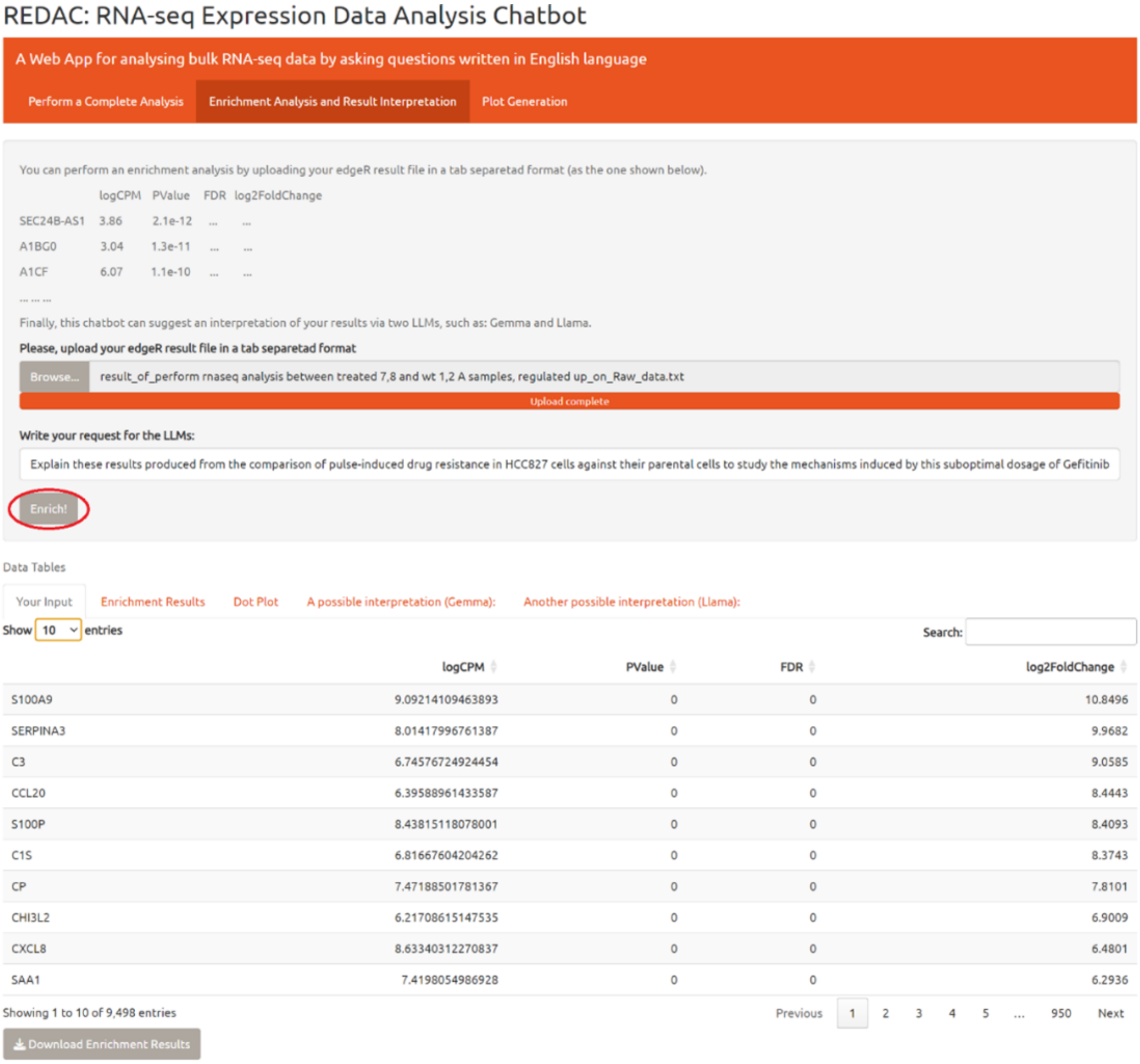

	logCPM	PValue	FDR	log2FoldChange
SEC24B-AS1	3.86	2.1e-12	...	...
A1BG0	3.04	1.3e-11	...	...
A1CF	6.07	1.1e-10	...	...
... ...				

	logCPM	PValue	FDR	log2FoldChange
S100A9	9.09214109463893	0	0	10.8496
SERPINA3	8.01417996761387	0	0	9.9682
C3	6.74576724924454	0	0	9.0585
CCL20	6.39588961433587	0	0	8.4443
S100P	8.43815118078001	0	0	8.4093
C1S	6.81667604204262	0	0	8.3743
CP	7.47188501781367	0	0	7.8101
CHI3L2	6.21708615147535	0	0	6.9009
CXCL8	8.63340312270837	0	0	6.4801
SAA1	7.4198054986928	0	0	6.2936

Figure 13: The enrichment analysis module interface showing the file upload functionality for edgeR differential expression results. Users upload their results file via the Browse button, with the first 10 lines displayed for verification. A contextual prompt is provided to guide LLM interpretation of the enrichment analysis. The example prompt reads: "Explain these results produced from the comparison of pulse-induced drug resistance in HCC827 cells against their parental cells to study the mechanisms induced by this suboptimal dosage of Gefitinib". Analysis is initiated using the Enrich! button (highlighted in red).

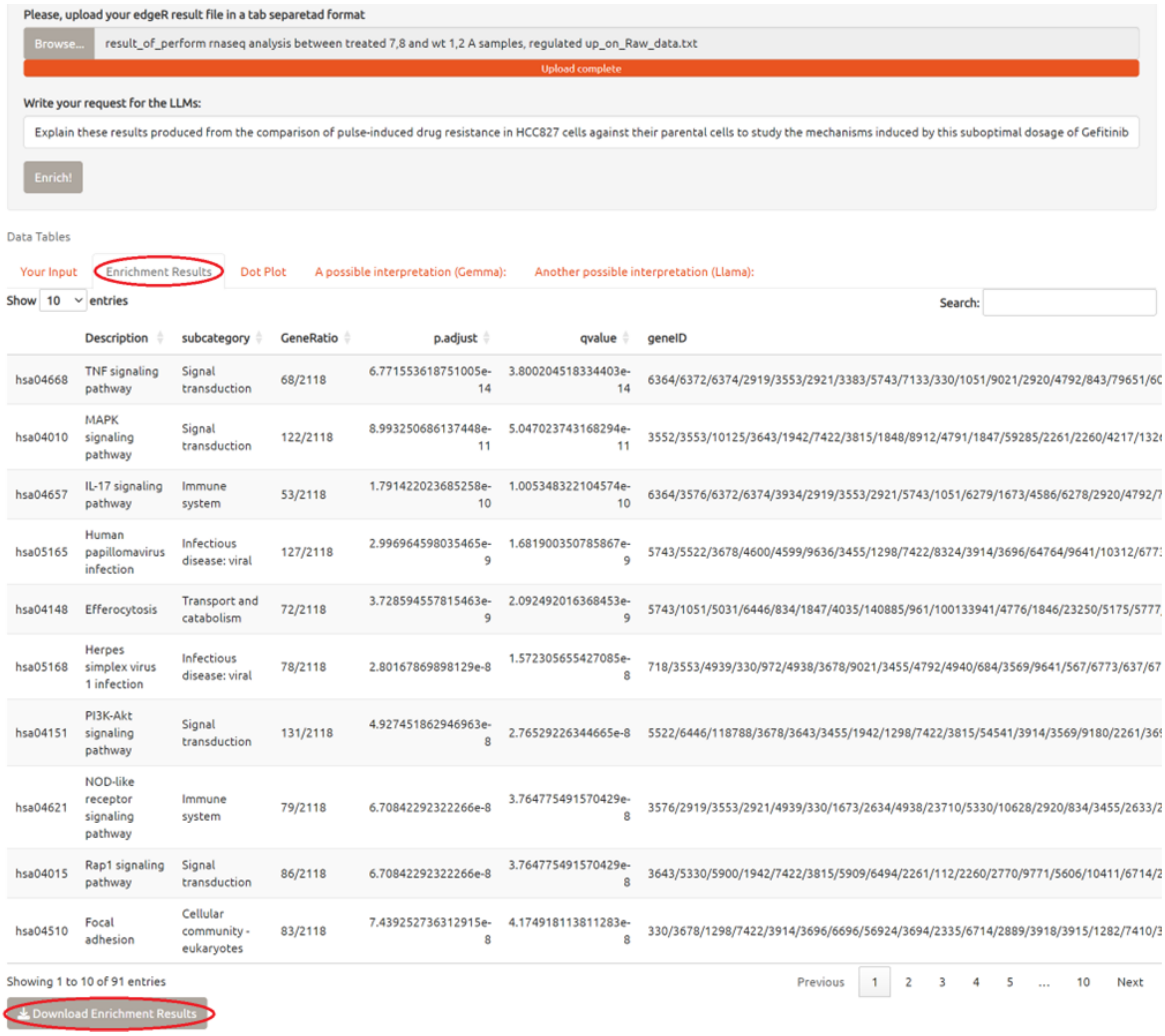

Figure 14: KEGG pathway enrichment analysis results interface. Upon completion of the analysis, users can access the enrichment results through the Enrichment Results tab (highlighted in red circle) to view the statistical output, and utilize the Download Enrichment Results button (highlighted in red circle) to export the complete results table for further analysis.

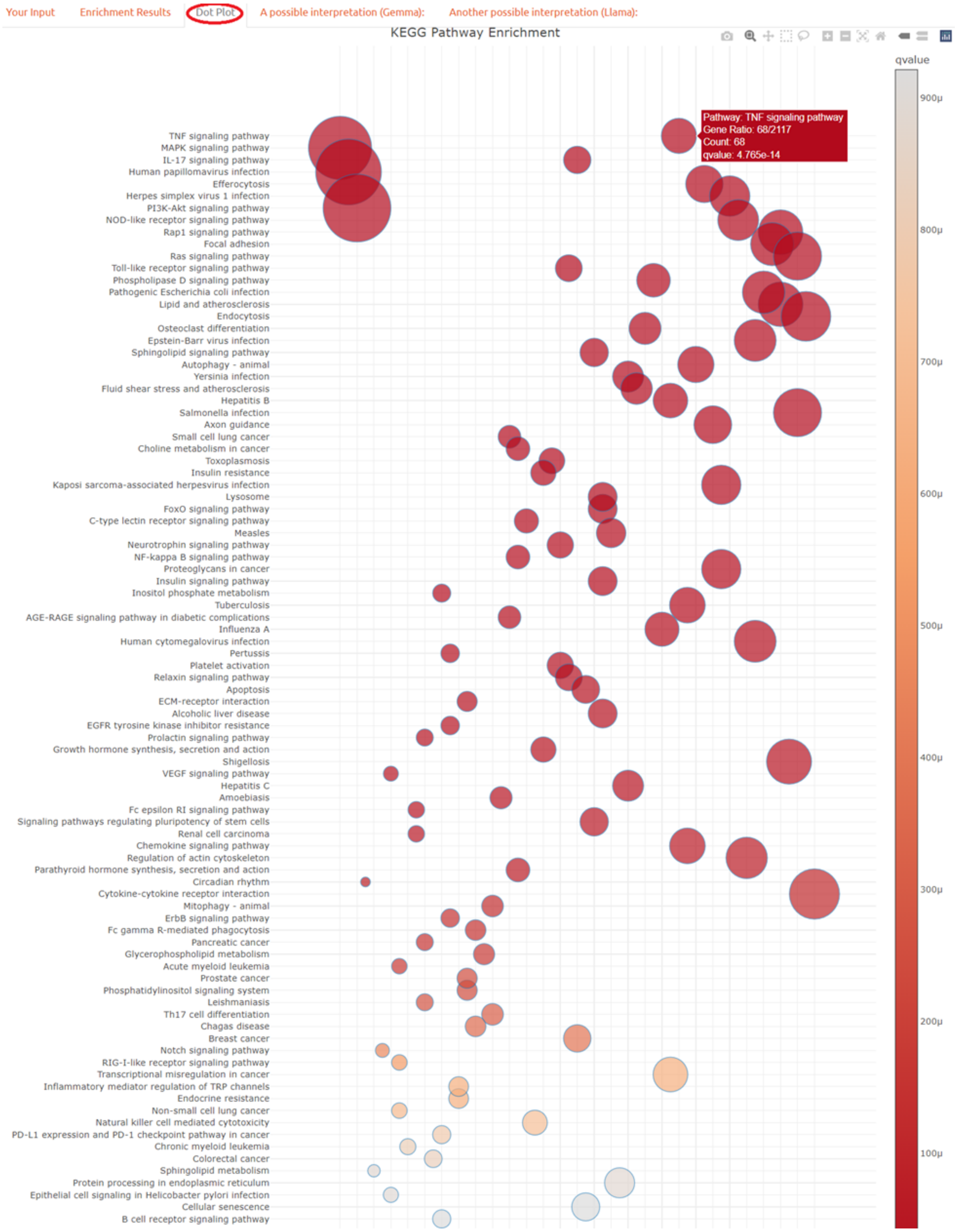

Figure 15: Selection of the Dot Plot tab (highlighted in red circle) generates a comprehensive dot plot visualization of the enriched pathways.

For biological interpretation of the enriched KEGG pathways, REDAC provides AI-powered analysis through two complementary large language models. Users can access the primary interpretation by selecting the *"A possible interpretation (Gemma)"* tab (Figure 16). This feature

leverages the Gemma model to generate contextual biological insights based on the enrichment results and the experimental context provided during the initial analysis setup.
For comparative analysis or alternative perspectives, users can obtain a secondary interpretation by selecting the “Another possible interpretation (LLaMA)” tab (Figure 17).

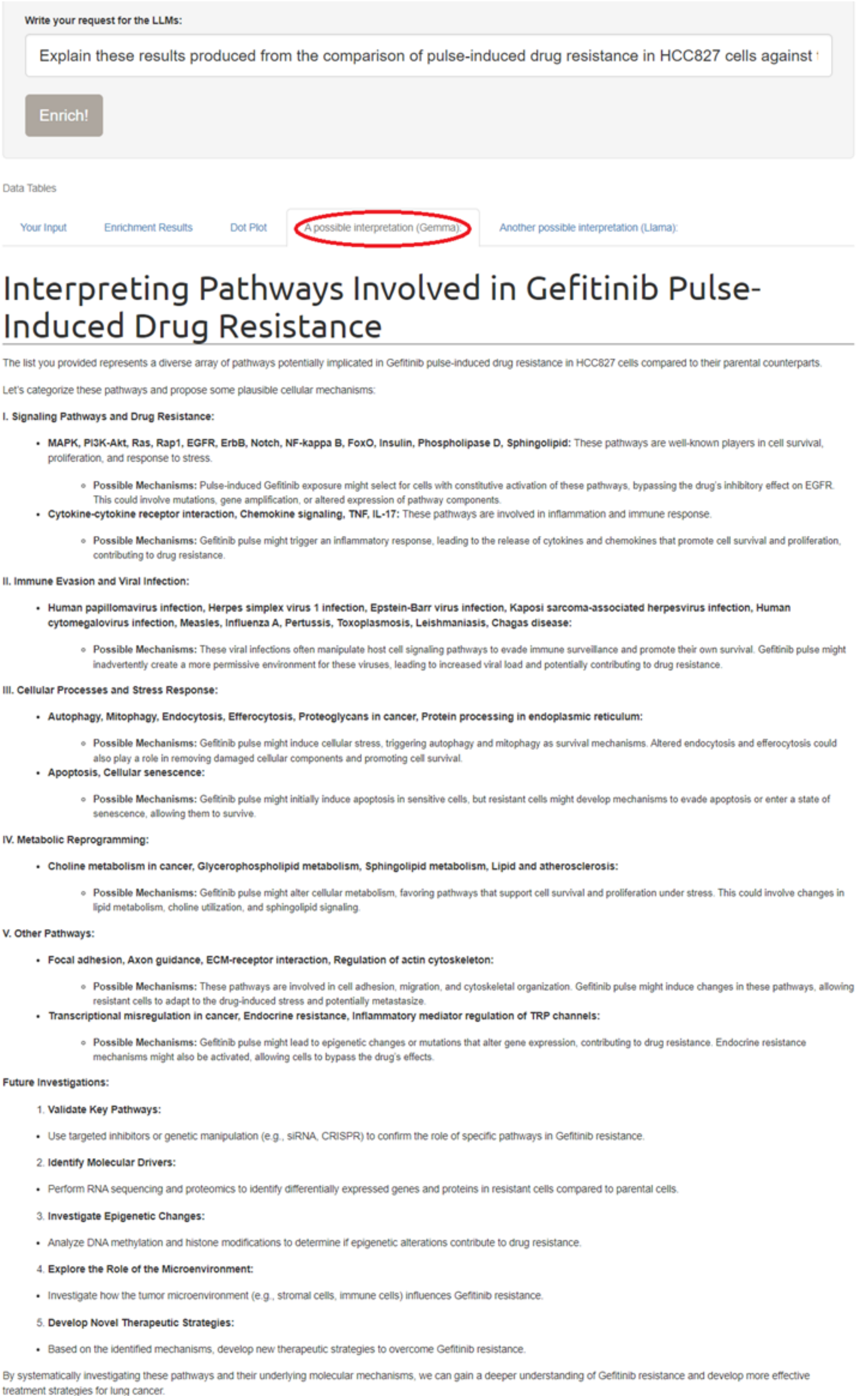

Figure 16: The Gemma-powered interpretation interface accessible through the “A possible interpretation (Gemma)” tab provides contextual biological analysis of the enriched KEGG pathways.

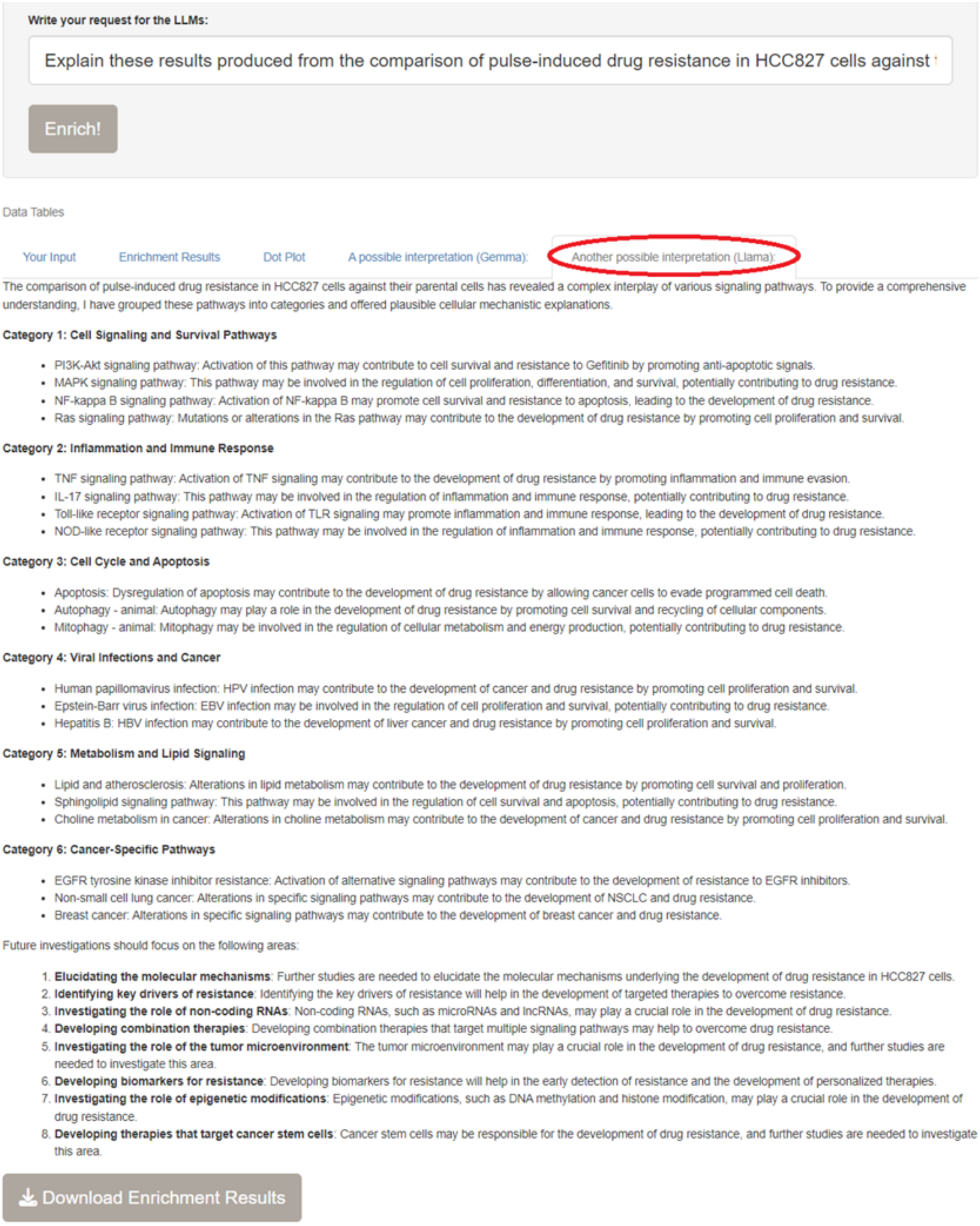

Figure 17: Alternative pathway interpretation using LLaMA can be accessed through the "Another possible interpretation (LLaMA)" tab (highlighted in red circle), enabling comparative analysis of enrichment results.

4 How to use the Plot Generation module

In this module enables users to create individual visualizations independently of the complete analysis workflow. This module supports two distinct input types: raw count matrices and differential expression results from edgeR analysis.
For count matrix input files, the following visualization types are available:

- boxplot,

- violin plot,
- heatmap,
- correlation heatmap,
- principal component analysis (PCA),
- three-dimensional PCA (3D PCA),
- hierarchical clustering dendrogram,
- density plot,
- PCA component loadings,
- gene interaction network,
- surface plot.

For edgeR differential expression result files, the module provides:

- volcano plot,
- MA plot,
- dot plot,
- KEGG pathway network (KEGGnet).

To demonstrate the network visualization tool, Figure 18 presents a gene interaction network generated from expression data in the input file Raw_data.txt. The analysis was initiated by entering "create a network" in the request field, which automatically triggered integration with the STRING database (`https://string-db.org/`). The STRING database provides seven categories of interaction evidence: text mining, experimental validation, curated databases, co-expression analysis, chromosomal neighborhood, gene fusion events, and phylogenetic co-occurrence.
Our implementation focuses exclusively on experimentally validated interactions, as these represent high-confidence physical protein-protein interactions suitable for integration with transcriptomic data. The STRING experimental interaction data are pre-integrated within REDAC, eliminating the need for manual data upload. To ensure analytical rigor, we applied a stringent confidence threshold, selecting only interactions with scores exceeding 980 (representing high-confidence associations). For each gene pair connected through such validated experimental evidence, we computed Pearson correlation coefficients using their respective expression profiles. Gene pairs exhibiting zero variance, which would result in undefined correlation values, were systematically excluded from the analysis.
The resulting network visualization employs a color-coded edge system where red edges denote positive correlations and blue edges indicate negative correlations. Edge color intensity corresponds to correlation strength: deep red indicates strong positive correlation, while intense blue represents strong negative correlation.
This interactive network visualization facilitates detailed exploration of gene co-expression patterns, enabling users to identify clusters of coordinately expressed genes and anti-correlated gene pairs that exhibit inverse expression relationships. Such network-based approaches can reveal regulatory relationships and functional modules that may not be apparent through conventional

pathway enrichment analyses, thereby providing complementary insights into the underlying biological systems by leveraging experimentally validated protein interaction data from the STRING database.

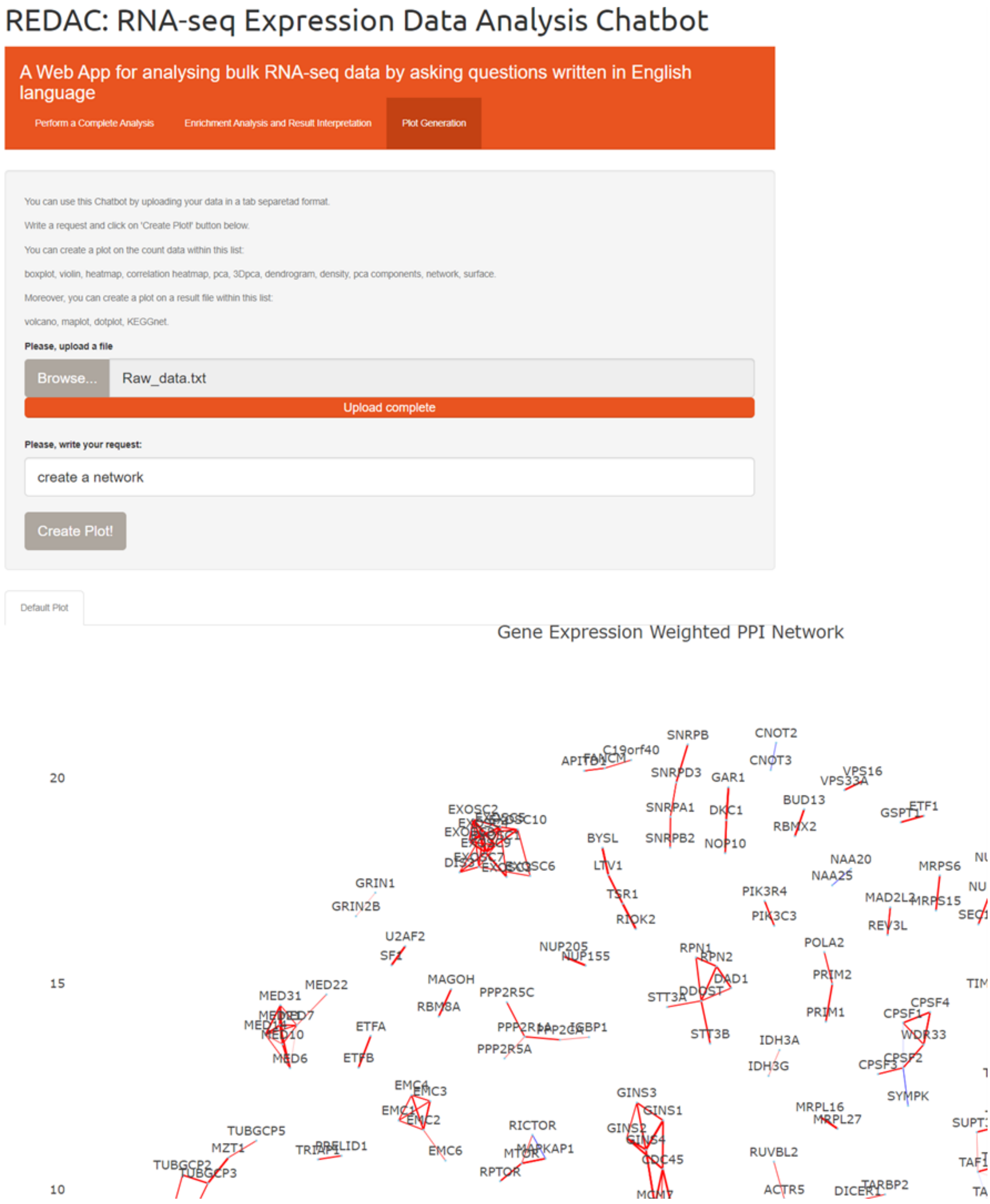

Figure 18: Gene interaction network integrating expression data with experimentally validated protein-protein interactions from the STRING database. Edge colors indicate expression correlations: red (positive), blue (negative), with intensity reflecting correlation strength.
